# Structural insights into a conserved mechanism of choline translocation through CHT

**DOI:** 10.1126/sciadv.aec1241

**Published:** 2026-05-29

**Authors:** Jesus Vilchez-Garcia, Adrián Martínez-Jiménez, Hanxing Jiang, Miguel Luengo, Borja Ochoa-Lizarralde, Jorge Pedro López-Alonso, Jerónimo Pérez-Lorente, Paola Bartoccioni, Raúl Estévez, Victor Guallar, Ekaitz Errasti-Murugarren, Iban Ubarretxena-Belandia, Igor Tascón

**Affiliations:** ^1^ Instituto Biofisika (UPV/EHU-CSIC), Leioa, Spain.; ^2^ Department of Biochemistry and Molecular Biology, University of the Basque Country (UPV/EHU), PO Box 644, 48080 Bilbao, Spain.; ^3^ Physiological Sciences Department, Institute of Neurosciences, School of Medicine and Health Sciences, University of Barcelona, Bellvitge Campus, L’Hospitalet de Llobregat, Spain.; ^4^ Electronic and Atomic Protein Modelling Group, Barcelona Supercomputing Center, Plaça d’Eusebi Güell, 1-3, E-08034 Barcelona, Spain.; ^5^ PhD program in Biotechnology, Faculty of Pharmacy and Food Sciences, University of Barcelona, 08028 Barcelona, Spain.; ^6^ Basque Resource for Electron Microscopy, Leioa, Spain.; ^7^ Institute for Research in Biomedicine (IRB Barcelona), The Barcelona Institute of Science and Technology (BIST), Baldiri Reixac 10, E-08028 Barcelona, Spain.; ^8^ The Spanish Center of Rare Diseases (CIBERER U-731), ISCIII, Madrid, Spain.; ^9^ Catalan Institution for Research and Advanced Studies (ICREA), Barcelona, Spain.; ^10^ Ikerbasque Foundation for Science, Bilbao, Spain.

## Abstract

Choline is an essential nutrient critical for cellular homeostasis across all domains of life. In humans, choline uptake in cholinergic neurons for its recycling into acetylcholine is mediated by the high-affinity Na^+^-dependent transporter SLC5A7 (CHT1). Prokaryotes also depend on choline as an osmo-protectant and as metabolite, raising the possibility that bacteria also have choline transporters akin to CHT1. Here, we identify and characterize a bacterial Na^+^-dependent choline transporter (sfCHT) with high sequence identity to CHT1. Cryo-EM structures of Na^+^- and choline-bound sfCHT reveal a LeuT-fold architecture with Na^+^ coordination geometry similar to CHT1. Captured in an inward-facing conformation, in sfCHT choline is found at a site near the cytoplasmic side. Computational analysis and transport assays of sfCHT and CHT1 variants reveal local conformational rearrangements in conserved residues along a defined pathway to the cytosolic site. These findings provide structural and mechanistic insights into intracellular choline transition, suggesting an evolutionarily conserved mechanism between the bacterial and human choline transporters.

## Teaser

Structural, computational, and functional analyses reveal a conserved choline intracellular transition in bacterial and human CHT.

## INTRODUCTION

Choline is a water-soluble quaternary ammonium cation that requires active transport to cross cell membranes and plays important structural and metabolic roles in prokaryotic (*1*) and eukaryotic cells (*2*). In humans, choline is a building block of phospholipids (*3*, *4*) and also a precursor of the neurotransmitter acetylcholine (ACh). In bacteria, choline is a structural constituent of some prokaryotic cell membranes (*4*) and serves as a precursor of osmo-protectants such as glycine betaine (*5*). In addition, choline can function as a sole source of carbon, nitrogen, and metabolic energy (*1*, *6*), and choline-derived metabolites can modulate virulence-associated gene expression (*7*). Together, these roles underscore the importance of efficient and tightly regulated choline transport across biological membranes in both domains of life.

Among the different types of choline transporters in humans, the high-affinity Na^+^-dependent choline transporter SLC5A7 (also known as CHT1) stands out for its role in mediating the uptake of choline, required for its recycling into ACh, at the presynaptic membrane of cholinergic neurons (*8*). CHT1 is a Na^+^/choline symporter (*9*–*11*) that exploits the electrochemical gradient of Na^+^ ions to drive the transport of choline. Recent high-resolution structures of human CHT1 in the presence of choline, Na^+^, Cl^−^, and the inhibitors hemicholinium-3 (HC-3) and ML352 (*12*–*14*), have revealed the canonical LeuT fold architecture of the transporter, the nature of its ion-binding sites, and key aspects of substrate recognition. Despite these advances, the mechanism of choline transition from the substrate-binding site into the cytosol remains poorly investigated.

In bacteria, several choline transporters have been identified and, in some cases, structurally characterized (*15*–*20*). Nevertheless, a substantial fraction of bacterial choline transporters remains unexplored. Through sequence analysis, we have identified homologous sequences to the choline transporter CHT1 across bacterial genomes, with some approaching 40% amino acid sequence identity. However, it remains speculative whether these proteins are capable of choline transport or share any structural and mechanistic determinants with CHT1.

Here, we identify and characterize a Na^+^-dependent choline transporter from *Salimicrobium flavidum* (sfCHT), a bacterial homolog of human CHT1. We demonstrate that sfCHT mediates Na^+^-dependent choline transport and determine cryo–electron microscopy (cryo-EM) structures of Na^+^- and choline-bound sfCHT. These structures reveal a conserved LeuT-fold architecture and capture sfCHT in an inward-facing conformation with choline positioned near the cytoplasmic exit. Integrating cryo-EM structural analysis with molecular simulations and choline uptake assays, we delineate the sequential local conformational changes that guide choline from its binding site toward the cytosol. Functional analyses of conserved pathway residues in both sfCHT and human CHT1 further demonstrate that intracellular choline transition relies on a shared, evolutionarily conserved mechanism across bacterial and human transporters.

## RESULTS

### sfCHT is a prokaryotic Na^+^-dependent choline transporter inhibited by HC-3

**Fig. 1.**
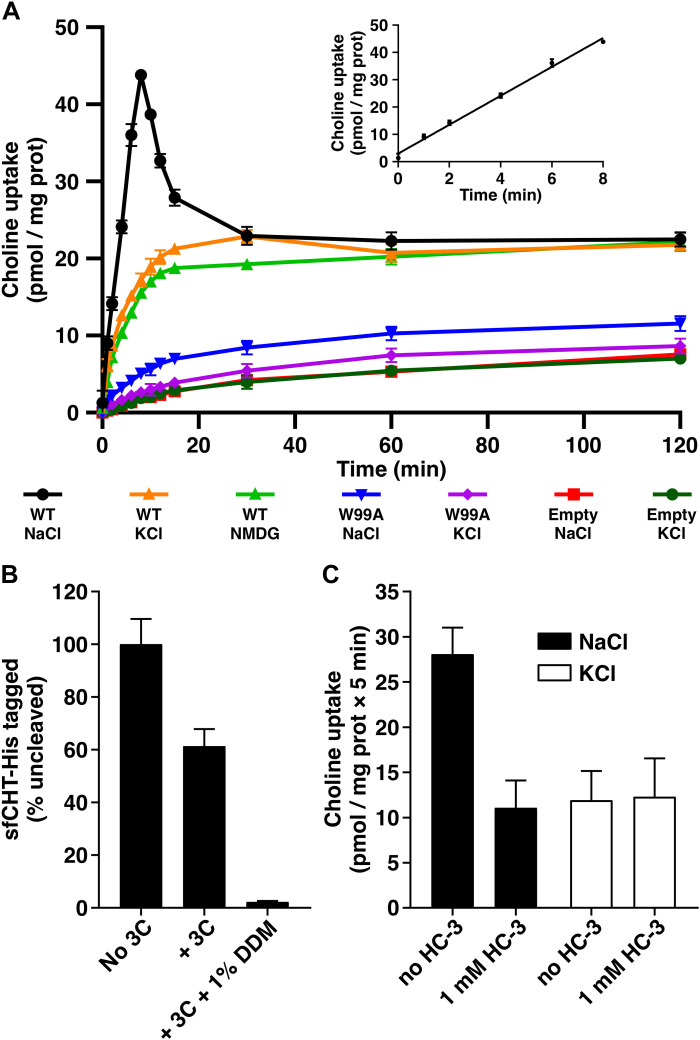
Transport activity of sfCHT reconstituted into *E. coli* polar lipids liposomes. (**A**) Time course (0 to 120 min) of 1 μM [^3^H]-choline (1 μCi/ml) uptake (pmol/mg protein) into WT sfCHT-PLs in a sodium-containing (137 mM NaCl, black circles) or sodium-free (137 mM KCl, orange triangles; 137 mM NMDG, green triangles) uptake buffer, into transport-defective W99A mutant sfCHT-PLs in a sodium-containing (137 mM NaCl, blue triangles) or sodium-free (137 mM KCl, purple diamonds) uptake buffer, and into empty liposomes in a sodium-containing (137 mM NaCl, red squares) or sodium-free (137 mM KCl, green circles) uptake buffer. Inset: Time course (0 to 8 min) of 1 μM [^3^H]-choline (1 μCi/ml) uptake (pmol/mg protein) into sfCHT-PLs in sodium-containing uptake buffer. Data correspond to a representative experiment, performed using three replicates. A second independent experiment gave similar results. (**B**) Random incorporation of the sfCHT protein in PLs. His-tagged sfCHT-PLs were treated with HRV-3C protease (3C) for 2 hours, at 4°C. The protease site is accessible in the inside-out His-tagged–inserted molecules, resulting in His tag cleavage. Protease-treated PLs were then mixed with loading sample buffer, analyzed in a 10% polyacrylamide gel, and densitometric results are shown. As a result, less than half of the WT proteins were inserted right-side-out when reconstituted in *E. coli* polar lipid liposomes. (**C**) 1 μM [^3^H]-choline (1 μCi/ml) uptake (pmol/mg protein) into sfCHT-PLs in the presence and absence of 1 μM HC-3 in sodium-containing and sodium-free (137 mM KCl) uptake buffers. Data (mean ± SD) are from three experiments with three replicates per condition.

We selected sfCHT from the halophilic bacterium *S. flavidum* following an expression and purification screening of prokaryotic homologs displaying a 34 to 38% amino acid sequence identity and a similar predicted topology with CHT1 (fig. S1). sfCHT was expressed in *Escherichia coli* as a C-terminal 3C-His_10_-tag fusion, solubilized in dodecyl-β-d-maltoside (DDM), and purified by a combination of Ni–nitrilotriacetic acid (NTA) and size exclusion chromatography (fig. S2). Functional reconstitution of sfCHT into proteoliposomes (PLs) revealed distinct transport behaviors depending on ionic conditions and transporter orientation. In the presence of an inwardly directed Na^+^ gradient, sfCHT mediated robust choline uptake characterized by a pronounced transient overshoot, consistent with concentrative Na^+^-dependent transport (Fig. 1A), as previously reported for liposome reconstituted CHT1 transporter (*21*). In contrast, when Na^+^ was replaced by *N*-methyl-d-glucamine (NMDG^+^) or K^+^, lower-level uptake persisted without an overshoot, consistent with transport mediated by inside-out–oriented sfCHT operating independently of extracellular Na^+^ (Fig. 1B). Empty liposomes and a transport-deficient mutant (W99A) failed to accumulate choline under all conditions tested, demonstrating that Na^+^-independent uptake reflects bona fide sfCHT activity rather than nonspecific permeation. Pharmacological inhibition with HC-3 (*9*), which targets the extracellular face of CHT1 (*12*–*14*), selectively suppressed Na^+^-dependent uptake but had no effect in Na^+^-free conditions (Fig. 1C), further supporting that choline transport in the absence of Na^+^ arises from inside-out–oriented transporters inaccessible to HC-3. Last, removal of K^+^ had minimal impact on Na^+^-independent uptake, indicating that potassium is not a critical coupling ion for choline transport by inside-out sfCHT. Together, these data establish sfCHT as a Na^+^-dependent choline transporter inhibitable by HC-3 while revealing an additional Na^+^-independent transport mode attributable to transporter orientation in the reconstituted system.

### LeuT-fold architecture of sfCHT

To elucidate the architecture of sfCHT, we collected cryo-EM images of the transporter in DDM micelles at pH 7.4 in the presence of 100 mM NaCl and in the absence of choline in our in-house 300 kV Krios G4 equipped with a BioContinuum energy filter and a K3 direct electron detector camera. Two-dimensional (2D) classification of the particles extracted from these images yielded a single ab initio class with clear discernible density for transmembrane (TM) helices that remain visible after the detergent micelle region fades out. 3D classifications using the good ab initio reconstruction and three decoy models as references followed by nonuniform and local refinements resulted in a 3D reconstruction at 2.83 Å nominal resolution (fig. S3 and Table 1).

**Table 1.** Cryo-EM structure determination. Cryo-EM data collection, refinement, and validation statistics.

	Na^+^-bound sfCHT (EMDB-54347) (PDB-9RWT)	Choline-bound sfCHT (EMDB-54346) (PDB-9RWS)
Data collection and processing		
Magnification	105,000	130,000
Voltage (kV)	300	300
Electron exposure (e^−^/Å^2^)	60.6	50.0
Defocus range (μm)	−1.0 to −1.6	−1.0 to −1.6
Pixel size (Å/pixel)	0.8238	0.6462
Symmetry imposed	C1	C1
Initial particle images (no.)	6,671,231	5,260,773
Final particle images (no.)	285,470	447,418
Map resolution (Å)	2.83	3.28
FSC threshold	0.143	0.143
Map resolution range (Å)	2.3 to 4.3	2.8 to 4.8
		
Refinement		
Initial model used (PDB code)	ModelAngelo model	9RWT
Model resolution (Å)	2.83	3.28
FSC threshold	0.143	0.143
Model resolution range (Å)	2.3 to 4.3	2.8 to 4.8
Map sharpening *B* factor (Å^2^)	−92.6	−149.7
Model composition		
Nonhydrogen atoms	3,895	3,903
Protein residues	512	512
Ligands	1	3
*B* factors (Å^2^)		
Protein	36.76	40.53
Ligand	24.10	62.51
RMSD		
Bond lengths (Å)	0.004	0.002
Bond angles (°)	0.614	0.501
Validation		
MolProbity score	1.55	1.35
Clashscore	8.87	5.69
Poor rotamers (%)	0.00	0.00
Ramachandran plot		
Favored (%)	98.23	97.83
Allowed (%)	1.77	2.17
Disallowed (%)	0.00	0.00

**Fig. 2.**
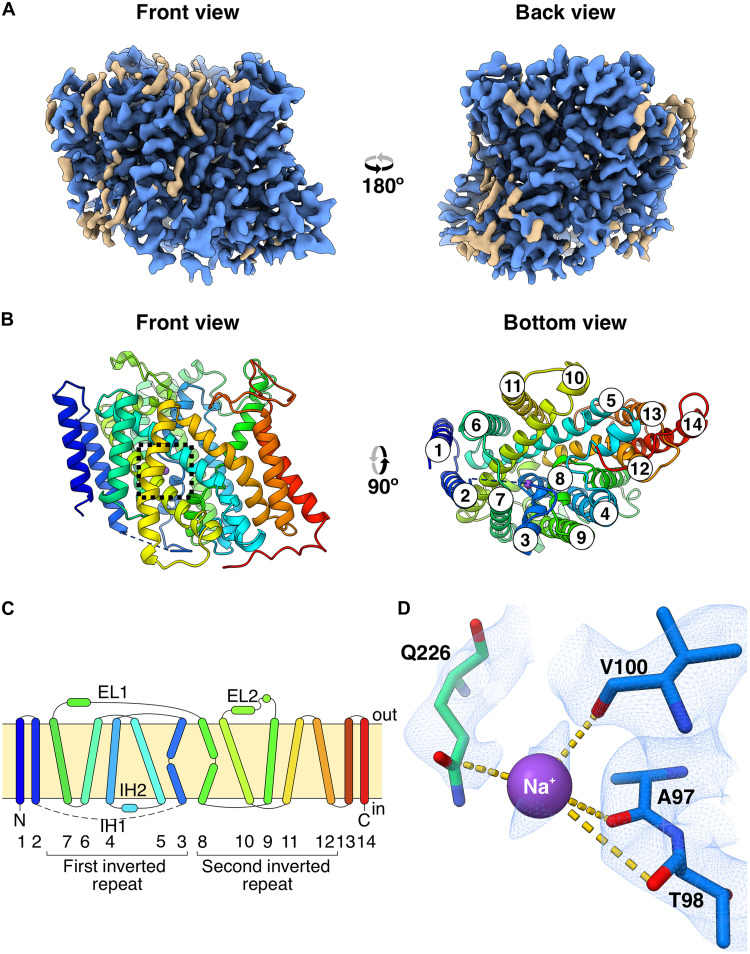
Overview of the structure of Na^+^-bound sfCHT. (**A**) Cryo-EM map of sfCHT at 2.83 Å nominal resolution. The front view has been arbitrarily set. The map regions colored in blue correspond to protein and ion-assigned densities, whereas nonprotein unassigned densities are brown colored. (**B**) Ribbon representation of the atomic model of sfCHT, colored in a rainbow gradient from blue (N terminus) to red (C terminus). The missing loop between TM helices 2 and 3 is depicted as a dashed line. Na^+^ cation is shown as a purple sphere. The rectangles in dashed lines highlight the location in the structure of the enlarged view of Na^+^ (D). The numbering of TM helices is shown in the bottom view (right). (**C**) Schematic representation of the topology of sfCHT, composed of 14 TM helices, organized in a 5 + 5 inverted structural repeats with two additional TM helices at the N terminus and two additional TMs at the C terminus. N, C, IH1, IH2, EL1, and EL2 denote the N terminus, C terminus, intracellular helix 1, intracellular helix 2, extracellular loop 1, and extracellular loop 2, respectively. The numbering of the TM helices is displayed below, with brackets indicating each of the inverted repeats. (**D**) Close-up view of the Na^+^ cation showing the coordination (dotted lines) by interacting residues. Cryo-EM density is displayed as a blue mesh.

The cryo-EM map of sfCHT captures the monomeric Na^+^-dependent choline transporter embedded in a DDM micelle (Fig. 2A, fig. S3, and Table 1). The cryo-EM map enabled near-complete de novo building of the sfCHT atomic structure (Fig. 2B and fig. S4), excluding the first 21 N-terminal and the last 7 C-terminal residues, and the 10 residues in between (70 and 80) the cytoplasmic loop linking TM helices 2 and 3. The 14 TM helices, with cytoplasmic N and C termini, adopt a canonical LeuT fold architecture. The N- (TM helices 3 to 7) and C-terminal domains (TM helices 8 to 12) form two inverted structural repeats related by pseudo-twofold symmetry relative to the membrane plane. These two domains constitute the core of the structure, and are flanked by N- and C-terminal hairpins of two TM helices each oriented toward the cytoplasm (Fig. 2C). Overall, the sfCHT structure in the absence of choline resembles the recently determined apo CHT1 [Protein Data Bank (PDB): 9BFI) (*13*) structures with a root mean square deviation (RMSD) of 1.074 Å for the 354 matched residues (fig. S5A).

LeuT-fold transporters typically harbor their substrate-binding sites within a central cavity built in between unwound segments of two pseudo-symmetrically related TM helices (*22*), which in sfCHT correspond to TM helices 3 and 8. TM helix 3 deviates from canonical α-helical geometry in a region that coincides with the hydrophobic core of the lipid bilayer at residues T98, W99, V100, G101, and G102, dividing the helix in two equally long TM3a and TM3b segments. Similarly, TM helix 8 unwinds at residues G294, G295, I296, P297, W298, and Q299, dividing the helix into a larger TM8a and a shorter TM8b segment (fig. S6). The resulting cavity in sfCHT harbors the putative choline-binding site lined by conserved aromatic and polar residues (W99, Y126, W176, W298, and W445), which display a similar configuration to that observed in apo CHT1 (*13*) (fig. S7A). The cavity appears constricted and lacks sufficient space to accommodate a choline molecule. This suggests that conformational rearrangements are required to enable choline binding at the substrate-binding site.

Near the central cavity, a cation density consistent with a Na^+^ ion was observed coordinated by residues A97, T98, and V100 in the unwound region of TM helix 3, and by Q226 in TM helix 7 (Fig. 2D and fig. S4B). The density was modeled as Na^+^ based on the buffer composition (100 mM NaCl), its coordination geometry, and its positional conservation relative to Na^+^ binding sites in LeuT-fold transporters. Notably, the location closely matches the Na^+^ site observed in the Na^+^/galactose transporter vSGLT (fig. S5B) and aligns with the observed Na^+^ position in choline-bound CHT1 structures (*12*–*14*).

The structure captures sfCHT in an inward-facing conformation, in which the extracellular side is tightly sealed by packing of TM helices 3b, 4, 5, and 12, while the intracellular side is open to the cytoplasm via an intracellular tunnel that provides sufficient space for choline (figs. S8A and S9). In contrast, the sfCHT AlphaFold model (AF-A0A1N7IZC0-F1) is closed to both sides of the membrane. We note that in this model the 10-residue cytoplasmic linker connecting TM helices 2 and 3a folds into a short intracellular helix (IH1) that blocks access to the intracellular tunnel from the cytoplasm (fig. S8B). Consistent with the cryo-EM structure representing an inward-facing conformation, in the cryo-EM map of sfCHT this cytoplasmic linker is disordered, which may facilitate the opening of the intracellular tunnel. In the inward-facing structures of CHT1 (*12*–*14*) the cytoplasmic loop is also unstructured, suggesting a conserved inward-facing opening mechanism.

### Inward-facing conformation of sfCHT with choline bound at a cytoplasmic site

In an effort to capture the binding mode of choline, we determined the cryo-EM structure of sfCHT at 3.28 Å nominal resolution in the presence of sodium and 1 mM choline (figs. S10 and S11A and Table 1). The cryo-EM map allowed us to unambiguously build the atomic model for residues 22 to 69 and 81 to 543, as in the Na^+^-bound structure (fig. S11). Cryo-EM captures sfCHT in the same inward-facing conformation as the Na^+^-bound structure (RMSD of 0.423 Å for the 512 built residues), with a Na^+^ ion found at the same conserved position at the intracellular tunnel near the central cavity comprising the substrate-binding site within TM helices 3 and 8 (figs. S11C and S12B). Nevertheless, this site presents a local tightening that allows us to build a water molecule, which was not observed in the Na^+^-bound structure.

**Fig. 3.**
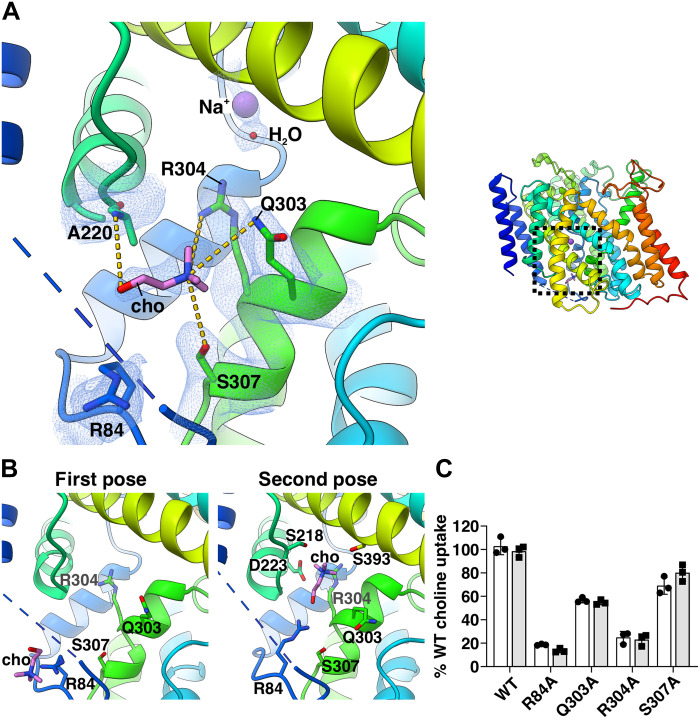
Interaction of choline and sfCHT. (**A**) Close-up view of the highlighted region in the experimentally determined choline-bound sfCHT structure, illustrating choline positioned at the cytoplasmic site. The surrounding cryo-EM density is displayed as a blue mesh, and interactions between choline and coordinating residues are indicated by yellow dashed lines. (**B**) Close-up view of two consecutive choline poses in PELE-predicted models. Residues located within interaction distance of choline and gating residues lining the intracellular tunnel are displayed in sticks. (**C**) Transport activity of R84A, Q303A, R304A, and S307A sfCHT variants reconstituted in liposomes. One micromolar (white bars) and 50 μM (gray bars) [^3^H]-choline (1 μCi/ml) 5 min uptake into PLs carrying WT and variant sfCHT is shown. Data are normalized to WT sfCHT choline uptake activity. Data correspond to three experiments with three biological replicates per condition.

We could not identify any density for choline within this cavity, which as in the Na^+^-bound structure was hindered by the side chains of the aromatic and polar residues (W99, Y126, W176, W298, and W445). Instead, we found nonprotein density consistent with choline close to the cytoplasmic exit of the intracellular tunnel (Fig. 3A and figs. S12C and S13). In this site, the trimethylammonium headgroup of choline interacts with the side chain of residues R304, Q303, and S307 from TM helix 8a, while the hydroxyl moiety establishes a hydrogen bond with the backbone amide of residue A220 in TM helix 7 (Fig. 3A). The close proximity of choline to the cytoplasmic surface of the membrane suggests we may have identified a cytoplasmic site. Consistent with this hypothesis, alanine substitutions at Q303, R304, and S307 exhibited a marked reduction in choline uptake compared to wild-type (WT) sfCHT in liposome-based transport assays, without affecting either protein expression or liposome reconstitution efficiency (Fig. 3C and fig. S14A). Together, the specific molecular contacts observed in the structure and the loss-of-function phenotypes of the corresponding variants support the assignment of choline at the identified cytoplasmic site. Notably, a comparable choline-binding cytoplasmic site has not been reported in available structures of CHT1 (*12*–*14*).

To further assess the relevance of the cytoplasmic site in the mechanism of choline transition along the intracellular tunnel, we performed PELE (protein energy landscape exploration) analysis, a Monte Carlo (MC) software that has shown excellent performance in elucidating substrate migration mechanisms in membrane proteins (*23*–*25*). The PELE analysis was carried out using the highest resolution cryo-EM structure of sfCHT (Na^+^-bound structure) without any ion and commenced with an unsupervised docking of choline from outside of the transporter to explore different poses. PELE predicted two minimum-energy poses for choline at the exit of the intracellular tunnel, in an arrangement that closely resembles the choline position observed in the cryo-EM map that represents an intermediate between both predicted poses (Fig. 3B). Notably, while R84, located in the structured part of the cytoplasmic linker connecting TM helices 2 and 3a, appears in an extended conformation in the second PELE-predicted pose, in the experimentally determined sfCHT structure and in the first PELE-predicted pose, R84 adopts a bent conformation (Fig. 3, A and B). This bent conformation creates space for choline. The substitution R84A in sfCHT resulted in a notably impaired choline uptake activity, indicating that this residue may contribute to choline translocation (Fig. 3C and fig. S14A). We suggest R84 plays a key role in the final step of substrate release to the cytoplasm. In both the cryo-EM structure and the second minimum-energy pose, choline is positioned immediately after Q303 and R304 (Fig. 3), implicating these residues as potential gating elements that prevent backflow of the substrate into the intracellular tunnel.

### Structural basis of choline transition from the substrate-binding site into the cytosol

**Fig. 4.**
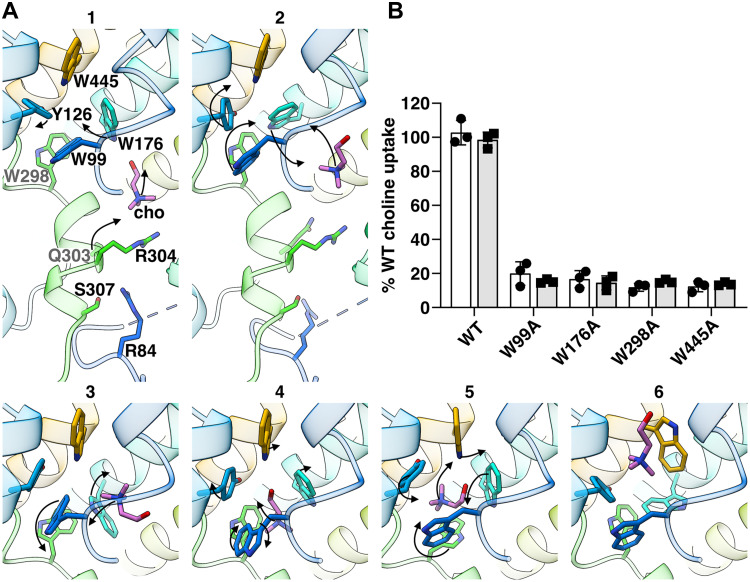
Local conformational changes along the translocation pathway. (**A**) Sequential PELE-predicted poses toward the substrate-binding site. Panels 1 and 2 provide a view of the translocation pathway, whereas panels 3, 4, 5, and 6 focus on the rearrangements within the substrate-binding site. Side chains displayed as sticks represent PELE-identified key residues in choline-binding and translocation. Arrows indicate the direction of the movement of choline and side chains to the next pose. (**B**) Transport activity of W99A, W176A, W298A, and W445A sfCHT variants reconstituted in liposomes. One micromolar (white bars) and 50 μM (gray bars) [^3^H]-choline (1 μCi/ml) 5 min uptake into PLs carrying WT and variant sfCHT is shown. Data are normalized to WT sfCHT choline uptake activity. Data correspond to three experiments with three biological replicates per condition.

Starting from the pose at the exit of the intracellular tunnel toward the central cavity, PELE simulations identified additional minimum-energy poses along the tunnel and within the substrate-binding site that help identify the structural determinants underlying choline transit from the substrate-binding site to the cytoplasmic vestibule where choline was observed in the cryo-EM structure. Two intermediate poses of choline within the intracellular tunnel (Fig. 4A, poses 1 and 2) revealed conformational changes of the side chains of residues Q303 and R304, which would permit the passage of choline, supporting their role as gating residues regulating exit from the intracellular tunnel into the cytoplasmic vestibule. Consistently, in vitro functional characterization of Q303A and R304A variants resulted in a complete loss of choline uptake (Fig. 3C and fig. S14A).

PELE analysis also predicted a minimal-energy pose at the canonical substrate-binding site within the unwound regions of TM helices 3 and 8 (Fig. 4A, pose 5), corresponding to an occluded state (fig. S8C), as reported for other LeuT-fold transporters, including CHT1 (*12*, *13*, *24*–*27*). In the predicted substrate-binding site pose, the trimethylammonium headgroup of choline forms π-cation interactions with the indole of W176 and W298 in TM helices 5 and 8, respectively, while the substrate hydroxyl group interacts with the imidazole ring of W99 and W445 in TM helices 3 and 12, respectively. In addition, PELE analysis suggests that conformational flexibility of the side chains of residues W99 and W176 is necessary for choline transition toward the intracellular tunnel (Fig. 4A, poses 3 and 4), while W445 might also act as an extracellular gate (Fig. 4A, pose 6). In support of this, liposome-based transport assays of W99A, W176A, W298A, and W445A sfCHT variants showed a near-complete loss of choline uptake activity, consistent with essential roles at the binding site (Fig. 4B). The calculated choline pose observed in our PELE analysis closely resembles that of the substrate-bound conformations observed in CHT1 cryo-EM structures (*12*–*14*), although with substantial differences (fig. S7B). In CHT1, a Cl^−^ ion is observed within the substrate-binding site where it interacts with cavity-forming residues, accommodating them to optimally coordinate the choline molecule. Whether this Cl^−^ ion may play a role in substrate recognition and/or translocation in CHT transporters needs further investigation.

The identified gating residues and inferred choline progression are consistent with alternating-access models in LeuT-fold transporters (*28*, *29*). Coordinated local conformational rearrangements of residues W99, W176, W298, Q303, R304, and R84 appear to create transient pockets that guide choline through the intracellular tunnel (fig. S15A and movie S1). Molecular dynamics simulations further support this pathway, yielding a similar choline trajectory shaped by rearrangements of tunnel-lining residues (fig. S16). Unbiased simulations clearly show choline entering the tryptophan enclosed substrate binding site and staying there for the whole simulation, 2 μs, observing multiple π-cation interactions (fig. S16, A and B), following an analogous path to the one observed in PELE.

### An evolutionarily conserved intracellular pathway for choline transport

To assess whether this pathway is conserved in CHT1, we performed PELE simulations using apo CHT1 cryo-EM structure (PDB: 9BFI) (*13*). These simulations revealed a comparable intracellular trajectory mediated by transient pockets formed by conserved residues, and predicted a choline pose within the cytoplasmic vestibule consistent with the site observed in the sfCHT cryo-EM structure (fig. S15B). Together, these results suggest a conserved mechanism of choline transition from the substrate-binding site to the cytoplasmic site in bacterial and human transporters.

**Fig. 5.**
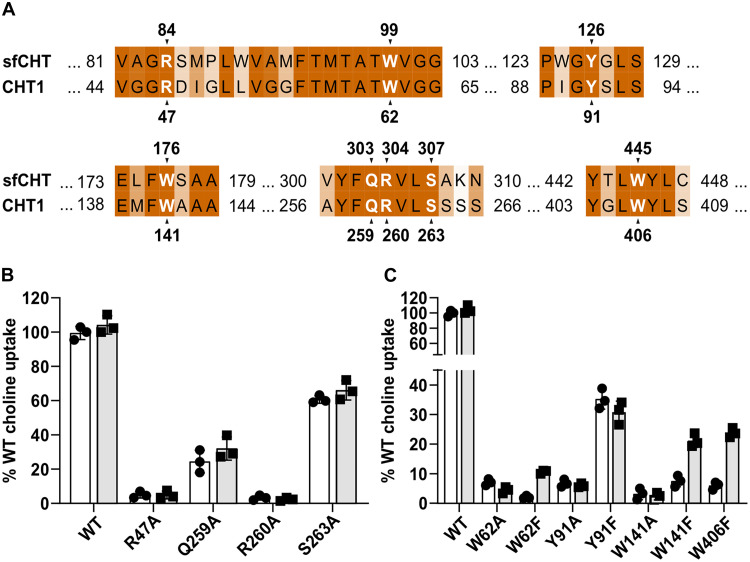
Intracellular pathway for choline transport is conserved in CHT1. (**A**) Sequence alignment between sfCHT and CHT1 showing that the key residues in sfCHT, identified by PELE and substitution analysis, are conserved in CHT1. Transport activity of selected CHT1 variants within the translocation pathway (**B**) or located at the substrate-binding site (**C**). Both panels show 1 μM (white bars) and 50 μM (gray bars) [^3^H]-choline (1 μCi/ml) uptake in HeLa cells by WT and variant CHT1. Uptake activity is normalized for WT CHT1 choline uptake. Data (mean ± SD) corresponds to three independent experiments run in quadruplicate (biological replicates). Endocytosis resistant CHT1 LV/AA variant, we refer as WT CHT1 in the context of this study, was used as template for the generation of CHT1 variants.

To experimentally test this hypothesis, we leveraged the high sequence identity between both transporters (Fig. 5A and fig. S1), and we carried out a targeted substitution analysis of conserved residues implicated in choline transition. Alanine substitutions in CHT1 at positions corresponding to sfCHT tunnel residues, *R47* (R84), *Q259* (Q303), *R260* (R304), and *S263* (S307) (hereby CHT1 numbering italicized and related sfCHT numbering in parentheses), resulted in choline uptake profiles closely matching those observed for the equivalent sfCHT variants (Figs. 5B and 4B), without affecting trafficking to the plasma membrane or staining levels (fig. S14B). These findings support a conserved functional role for these residues in choline translocation. Consistent with this interpretation, kinetic analysis of the *Q259A* (Q303) and *S263A* (S307) CHT1 variants revealed a reduction in *V*_max_ without a substantial change in apparent choline affinity (fig. S17 and Table 2), suggesting a role in substrate translocation rather than initial recognition.

**Table 2.** Kinetics parameters *K*_M_ and *V*_max_ derived from WT and variant CHT1 uptake of [^3^H]-choline. Data from three representative experiments and mean ± SD are shown.

	Mean ± SD	
	*K*_M_ (μM)	*V*_max_ (pmol/mg prot•min)
WT	12.17 ± 1.06	1181.15 ± 47.81
W62F	401.72 ± 32.24	627.40 ± 41.60
Y91F	22.23 ± 1.91	352.52 ± 36.69
W141F	270.81 ± 14.66	587.57 ± 27.92
W406F	357.60 ± 99.13	543.07 ± 61.01
Q259A	10.08 ± 1.50	386.82 ± 21.33
S263A	14.60 ± 2.03	659.43 ± 41.04

We next examined conserved aromatic residues within the substrate-binding site. Alanine substitution of *W62* (W99), *Y91* (Y126), *W141* (W176), *W254* (W298), and *W406* (W445) in CHT1 abolished choline uptake (*12*, *13*) without affecting trafficking to the plasma membrane or staining levels (Fig. 5C and fig. S14B), indicating that these residues are critical for transporter function. Substitution of selected aromatic residues with phenylalanine partially restored transport activity (Fig. 5C). Kinetic characterization of *W62F*, *W141F*, and *W406F* CHT1 variants revealed an approximately 10-fold decrease in apparent choline affinity accompanied by a ~50% reduction in *V*_max_, whereas the *Y91F* variant primarily affected *V*_max_ with minimal impact on apparent affinity (fig. S17 and Table 2). These results indicate that aromatic residues in the binding pocket contribute to both substrate recognition and efficient translocation.

Together, structural, computational, and functional analyses converge on a conserved mechanism of choline recognition and intracellular transition shared between sfCHT and human CHT1. These findings establish an evolutionarily conserved pathway guiding choline from the substrate-binding site to the cytosol in bacterial and human choline transporters.

## DISCUSSION

Choline is a structural component of prokaryotic cell membranes (*4*), and its uptake is a critical adaptive strategy in many bacterial species, enabling survival under osmotic stress (*5*), serving as carbon, nitrogen, and energy source (*1*, *6*), and acting as a virulence factor (*7*, *19*). In this study, we identify a prokaryotic Na^+^-dependent choline transporter from *S. flavidum* (sfCHT) inhibitable by HC-3 that shares high sequence identity with human CHT1. Cryo-EM structures of sfCHT in Na^+^- and choline-bound inward-facing conformations reveal a canonical LeuT fold architecture, Na^+^-binding sites similar to LeuT and other sodium-coupled symporters, as reviewed in (*28*), including CHT1 (*12*–*14*), and an unprecedented cytoplasmic choline-binding site. Integration of cryo-EM structures with computational modeling and transport assays provides mechanistic insights into how choline is released from the canonical substrate-binding site and guided toward the cytoplasm mediated by local conformational changes through a defined intracellular pathway. Comparative substitution analysis between sfCHT and CHT1 reveals that key aspects of choline recognition and intracellular transition are evolutionarily conserved, advancing our understanding of CHT-mediated choline transport.

Secondary active transporters operate via an alternating access mechanism, transitioning between outward- and inward-facing states through intermediate occluded conformations (*28*, *29*). The sfCHT cryo-EM structures capture an inward-facing conformation with a tightly sealed extracellular side and a wide-open intracellular tunnel. In contrast, the AlphaFold model of sfCHT predicts a short helix (IH1) within the linker between TM helices 2 and 3 that blocks cytoplasmic access to the intracellular tunnel, resembling the outward-facing conformations of other SLC5 transporters such as CHT1, SGLT1, and SGLT2 (*12*, *13*, *30*). Notably, IH1 is unstructured in inward-facing CHT1 structures (*12*–*14*), suggesting that during inward transition, the unwinding of IH1 releases the intracellular ends of the TM helices, enabling intracellular tunnel formation and substrate passage. These findings support a conserved transition mechanism between outward and inward conformations between sfCHT and CHT1.

**Fig. 6.**
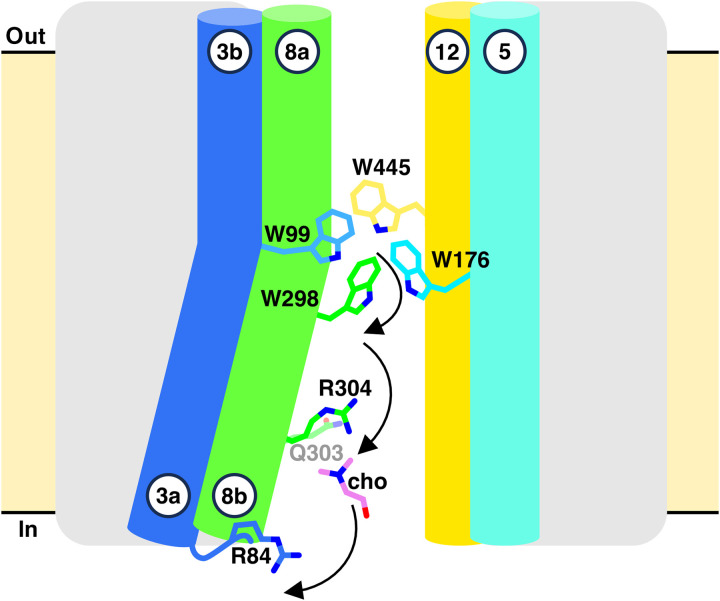
Model of choline transition from the substrate-binding site to the cytosol through CHT. Schematics of the inward-open cryo-EM structure of choline-bound sfCHT, with choline at the cytoplasmic site. Arrows indicate the stepwise movement of choline along the translocation pathway from the substrate-binding site to the cytoplasmic vestibule, inferred from the PELE-predicted poses. Choline and the residues involved in local conformational changes that facilitate the movement of the substrate are depicted as sticks.

The choline-bound sfCHT structure captures a stable intermediate state with choline bound to a cytoplasmic-facing site (Fig. 3). Together with PELE analysis, molecular dynamics simulations and transport assays of key sfCHT variants, these data support a stepwise mechanism of choline transition from the substrate-binding site into the cytoplasm (Fig. 6, fig. S15A, and movie S1). We posit that translocation initiates following partial release of choline from the binding site, facilitated by rearrangements of intracellular gate residues W99 and W176 (Fig. 4). Choline is then transiently stabilized between aromatic residues of the binding site and an intermediate gate formed by Q303 and R304 (Fig. 4). These residues likely serve a dual function: constraining the small choline molecule to prevent premature escape while retaining sufficient conformational flexibility to permit forward progression. Subsequent interaction with R84 promotes closure of the intermediate gate, stabilizing a prerelease state and limiting substrate back-diffusion (Fig. 3B). The bent conformation of R84 observed in the cryo-EM structure likely represents a late-stage intermediate preceding substrate release into the cytosol (Fig. 3A). Although S307 contributes to choline coordination at this site, its limited conservation among bacterial homologs (fig. S1) and modest functional impact suggest a supportive rather than essential role. Collectively, these observations are consistent with a multistep, residue-guided transition of choline through sfCHT (Fig. 6, fig. S15A, and movie S1).

This mode of substrate progression is conceptually consistent with steered molecular dynamics (SMD) studies of other secondary active transporters. In lactose permease (LacY), SMD simulations demonstrated that sugar translocation occurs through a series of metastable intermediates rather than a single concerted release event, with transient interactions between the substrate and specific residues progressively guiding movement along the transport pathway (*31*). Similarly, SMD analyses of the γ-aminobutyric acid (GABA) transporter revealed that substrate release toward the cytoplasm is governed by sequential rearrangements of intracellular gating residues, which generate transient stabilization points along the exit pathway (*32*). These studies emphasize that substrate transport in secondary active transporters is controlled by coordinated local dynamics that lower energetic barriers through stepwise progression. The intermediate choline-binding site and transient gating rearrangements observed in sfCHT are consistent with this broader mechanistic framework.

In the inward-facing sfCHT structure in the presence of choline, no substrate density was observed at the canonical binding-site between the unwound segments of TM helices 3 and 8, likely due to steric constraints imposed by cavity-lining residues in this conformation (fig. S7A). In contrast, CHT1 structures capture choline bound at this site in an occluded state (*12*–*14*). We cannot exclude that the DDM detergent micelle, used for sfCHT purification, stabilizes the inward-facing conformation and disfavors substrate occupancy at the canonical binding site, favoring binding at the cytoplasmic site. Nevertheless, PELE simulations identified a low-energy choline pose consistent with the canonical binding site observed in CHT1 (fig. S7B), indicating that sfCHT retains the structural capacity for substrate binding at this position. In CHT1, Cl^−^ has been proposed to stabilize the substrate-binding site, potentially accounting for differences in binding pose and conformational preference. The absence of Cl^−^ density in inward-facing sfCHT and apo CHT1 structures suggests that Cl^−^ may dissociate during substrate release, leaving a constricted binding site incompatible with choline binding. Whether Cl^−^ dependence represents a later evolutionary adaptation remains to be determined.

Although recent structural evidence has established the basis of substrate recognition in CHT1 (*12*–*14*), the mechanism by which choline transitions from the binding site to the cytoplasm remains unclear. PELE simulations of the apo inward-facing CHT1 structure reveal a choline trajectory along the intracellular tunnel toward the substrate-binding site that closely mirrors the pathway identified in sfCHT (fig. S15). Substrate progression is mediated by transient, metastable poses generated through local rearrangements of conserved residues, including *R47* (R84), *Q259* (Q303), *R260* (R304), and *S263* (S307). Notably, PELE predicts a choline pose in CHT1 that resembles the cytoplasmic site captured in the sfCHT cryo-EM structure, supporting the existence of a conserved prerelease intermediate. Guided by these simulations and sfCHT homology, targeted mutagenesis of conserved CHT1 residues revealed functional roles analogous to those observed in sfCHT (Fig. 5). Alanine substitution within the canonical binding site abolished transport, whereas conservative substitutions partially preserved activity, underscoring the importance of steric bulk for productive substrate positioning, as previously reported for other LeuT fold transporters (*24*–*26*, *33*). In contrast, substitutions of residues lining the intracellular tunnel primarily reduced transport capacity (*V*_max_) without affecting apparent affinity (*K*_m_), indicating roles in substrate translocation rather than recognition. Together, these results support a conserved, stepwise choline transition mechanism in which coordinated local conformational changes, rather than large-scale rearrangements, guide substrate release in both bacterial and human CHT transporters.

While CHT1 is well-established as the neuronal choline transporter supporting ACh biosynthesis, the physiological roles of its prokaryotic homologs remain unclear. In halophilic bacteria such as *S. flavidum* (*34*), a Na^+^-dependent choline transporter could efficiently exploit high extracellular Na^+^ to promote osmoadaptation (*35*), while also providing a source of carbon and nitrogen, as reported for *Pseudomonas aeruginosa* (*1*). This dual benefit may explain the retention of a CHT-type transporter as a general choline uptake system in bacteria. In contrast, higher eukaryotes rely primarily on facilitative, Na^+^-independent carriers such as FLVCR1 and FLVCR2 (*36*–*38*), whereas CHT1 has become specialized for high-affinity Na^+^-dependent uptake in neurons.

In summary our structural, computational, and functional analyses demonstrate that key aspects of the choline recognition and release into the cytosol are evolutionarily conserved between bacterial sfCHT and human CHT1. By revealing a stepwise substrate transition mechanism guided by transient interactions and local conformational rearrangements, paralleling principles established by SMD studies of LacY and the GABA transporter, this work places CHT-mediated choline transport within a broader framework of secondary active transport. These findings suggest that the fundamental principles underlying high-affinity Na^+^-dependent choline transport emerged early in evolution and were subsequently adapted to meet the specialized demands of the nervous system.

### Limitations of this study

While our analyses provide detailed insight into choline translocation from the substrate-binding site to the cytoplasm, a complete understanding of the CHT transport cycle remains limited by the lack of outward-facing structures and full characterization of ion coupling. In particular, the roles of Na^+^ and Cl^−^ in transport, such as the sequence of ion binding and release, or whether Cl^−^ is required in bacterial homologs, remain unresolved. The occupancy of the Na^+^-binding site in our inward-facing sfCHT structures may reflect stabilization of the ion site by the high Na^+^ concentration used during sample preparation, leaving open questions regarding the timing of ion coordination relative to choline translocation. Likewise, in the absence of an outward-facing structure, the mechanism by which choline entry into the binding site cannot be directly addressed. These limitations do not affect our interpretation of choline recognition and cytosolic release, but highlight important directions for future studies aimed at defining the full transport cycle and ion-coupling mechanisms.

## MATERIALS AND METHODS

### Sequence alignment

The sequence conservation of SLC5A7 was assessed using a list composed of *Homo sapiens* and 10 bacterial homologous sequences obtained from the BLAST-NCBI server. *S. flavidum*, *Pseudobacteriovorax antillogorgiicola*, *Cyclobacterium halophilum*, *Halopolyspora algeriensis*, *Algoriphagus chordae*, *Algoriphagus aquimarinus*, *Bacillus aryabhattai*, *Halobacillus aidingensis*, *Melghirimyces thermohalophilus*, and *Marininema mesophilum*. PROMALS3D (*39*) was used for multiple sequence alignment, enabling alignment based on both sequence and secondary structure prediction. Jalview (*40*) was used for figure preparation.

### Homologs selection

The selected bacterial sequences were codon optimized for expression, synthesized, and cloned into two types of pB24 expression vector by GenScript company, adding an N-terminal or a C-terminal His_10_ tag to the expressed protein, respectively, and transformed into *E. coli* BL21 cells. Cells were grown at 37°C in 5 ml LB medium supplemented with ampicillin (100 μg/ml), and overexpression was induced at different optical densities at 600 nm (OD_600_) by the addition of different amounts of arabinose ranging from 0.001 to 0.02% at 37° or 20°C, and at different induction times. Afterward, cells were harvested and resuspended in 20 mM tris-HCl (pH 7.4), 150 mM NaCl buffer, supplemented with 0.5 mM phenylmethylsulfonyl fluoride (PMSF), 1 mM benzamidine, and deoxyribonuclease (DNase) I. Cells were disrupted using sonication, and all subsequent steps were performed at 4°C. Cell debris and unbroken cells were pelleted at 15,000*g* for 15 min. Then, the membrane pellet was resuspended in 20 mM tris-HCl (pH 7.4), 150 mM NaCl buffer to a concentration of 100 mg/ml and solubilized by the addition of 1% DDM for 1 hour. Afterward, the solution was centrifuged at 180,000*g* for 30 min to pellet unsolubilized particles. The supernatant was incubated with Ni-NTA beads for 1 hour in 20 mM tris-HCl (pH 7.4), 150 mM NaCl, 0.04% DDM, and 10 mM imidazole buffer. Unbound and nonspecifically bound proteins were eluted with 20 mM tris-HCl (pH 7.4), 150 mM NaCl, 0.04% DDM, and 50 mM imidazole. The protein was further purified by size exclusion chromatography using a Superdex 200 Increase 10/300 GL column (GE Healthcare) previously equilibrated with 20 mM tris-HCl (pH 7.4), 150 mM NaCl, and 0.02% DDM. The expression tests were evaluated with SDS–polyacrylamide gel electrophoresis (PAGE) and nanoscale liquid chromatography–tandem mass spectrometry (nLC-MS/MS), and the best behaving targets were used for negative staining screening.

### Expression and purification of sfCHT

The gene encoding WT sfCHT was cloned into a pB24 expression vector, adding a C-terminal His_10_ tag to the expressed protein, and transformed into *E. coli* BL21 cells. An overnight preculture was grown at 37°C in LB medium supplemented with ampicillin (100 μg/ml). This culture was diluted 100-fold in 4 liters of fresh LB medium with ampicillin (100 μg/ml) at 37°C, and overexpression was induced by the addition of 0.01% arabinose when an OD_600_ of 1 was reached. After 2.5 hours of induction at 37°C, cells were harvested and resuspended in 20 mM tris-HCl (pH 7.4), 150 mM NaCl buffer, supplemented with 0.5 mM PMSF, 1 mM benzamidine, and DNase I. Cells were disrupted using an Emulsiflex-C5 high-pressure homogenizer (Avestin), and all subsequent steps were performed at 4°C. Cell debris and unbroken cells were pelleted at 15,000*g* for 15 min. Membranes were collected by centrifugation at 180,000*g* for 3 hours. The membrane pellet was resuspended in 20 mM tris-HCl (pH 7.4), 150 mM NaCl buffer to a concentration of 100 mg/ml and solubilized by the addition of 1% DDM for 1 hour. Afterward, the solution was centrifuged at 180,000*g* for 30 min to pellet unsolubilized particles. The supernatant was incubated with Ni-NTA beads for 1 hour in 20 mM tris-HCl (pH 7.4), 150 mM NaCl, 0.04% DDM, and 10 mM imidazole buffer. Unbound and nonspecifically bound proteins were eluted with 20 mM tris-HCl (pH 7.4), 150 mM NaCl, 0.04% DDM, and 50 mM imidazole. sfCHT was eluted by incubating the sfCHT-bound Ni-NTA beads with the protease 3C. The protein was further purified by size exclusion chromatography using a Superdex 200 Increase 10/300 GL column (GE Healthcare) previously equilibrated with 50 mM tris (pH 7.4), 100 mM NaCl, and 0.02% DDM. Fractions containing the protein sfCHT were pooled and concentrated for cryo-EM sample preparation. The purification process was assessed using SDS-PAGE and nLC-MS/MS.

### Negative staining

During the screening of homologous proteins of CHT1, those targets that could be purified were screened in negative staining for their suitability for cryo-EM: good particle distribution, without protein aggregates, and sufficient protein concentration. Specimens of sfCHT at different concentrations in 50 mM tris-HCl (pH 7.4), 100 mM NaCl, and 0.02% DDM buffer were incubated onto previously glow-discharged continuous carbon film 400-mesh copper grids for 2 min, then washed in buffer for 30 s and subsequently stained for 1 min with 2% (w/v) uranyl acetate at pH 5, previously filtered. The grids were blotted onto filter paper and loaded in a JEM 1400 Plus transmission electron microscope (JEOL Japan) operating at 100 kV with a scientific complementary metal-oxide semiconductor camera for image acquisition.

### Cryo-EM specimen preparation and data collection

Purified sfCHT was concentrated up to 4 mg/ml for cryo-EM sample preparation. In case of the choline-bound structure, right before vitrification, choline was added to a final concentration of 1 mM. Vitrification was carried out in a Thermo Fisher Scientific Vitrobot Mark IV double-side blotting automated plunge freezer at 4°C and 100% humidity. Three microliters of the sample were applied onto a previously glow-discharged UltrAuFoil R 0.6/1 gold foil on gold 300-mesh grid. The grid was double-side blotted for 3.5 to 5 s, using blot force 0, and was plunge-frozen in liquid ethane. The specimen was imaged on a 300 kV Krios G4 (Thermo Fisher Scientific), equipped with a BioContinuum/K3 camera (Gatan) operating in counting mode at a calibrated pixel size of 0.8238 Å/pix for Na^+^-bound sfCHT and 0.6462 Å/pix for choline-bound sfCHT. A defocus range between −1.0 and −1.6 μm was used, and movies were recorded with a maximum total accumulated exposure of 60 e^−^/Å^2^ fractionated into 60 frames in case of Na^+^-bound sfCHT or 50 e^−^/Å^2^ fractionated into 50 frames for choline-bound sfCHT. Movies were recorded automatically using EPU 2 (Thermo Fisher Scientific) with aberration-free image shift and fringe-free imaging.

### Image processing

Initial frame alignment of the recorded movies and contrast transfer function (CTF) estimation of the micrographs was carried out with cryoSPARC Live (*41*). A total of 18,603 and 21,999 micrographs were collected for Na^+^- and choline-bound sfCHT, respectively. Only micrographs with maximum resolution better than 3.8 Å according to CTF estimation were selected for further processing. Automatic picking in cryoSPARC Live was performed firstly without references by searching for Gaussian signals and then 2D classification helped to select good 2D class averages as templates for picking particles with reference. Subsequent data processing was carried out using cryoSPARC. This initial dataset contained 6,671,231 and 5,260,773 particles for Na^+^- and choline-bound sfCHT, respectively, both extracted to bin 2. Afterward, a harsh cleaning of particles was performed in 2D classification so only 2D class averages clearly showing TM helices were kept for further steps. These particles were used to build ab initio models with a starting resolution of 15 Å, a maximum resolution of 7 Å, 300 images per minibatch at the beginning and 1000 images at the end. We considered a good initial reference those volumes that keep cryo-EM density for TM helices after the density from the detergent micelle region fades out. Particles from discarded 2D class averages were used to build decoy models acting as initial references to trap bad particles to further clean the particle dataset during heterogeneous refinement. A total of 3,640,162 and 3,297,559 particles corresponding to Na^+^- and choline-bound sfCHT, respectively, were used together with the good ab initio model and three decoy models for two rounds of heterogeneous refinement, the first with an initial resolution of 10 Å and the second 8 Å. Afterward, a nonuniform refinement was carried out, starting with an initial resolution of 15 Å. To improve resolution, the selected particles so far were used as seeds for Topaz (*42*) training and extraction, and a similar protocol was followed with the extracted particles. After merging the previously curated particle set with those selected using Topaz and removing duplicate particles, two different approaches were used for Na^+^- and choline-bound sfCHT.

For Na^+^-bound sfCHT, an initial nonuniform refinement, using a dynamic mask including the micelle automatically generated by cryoSPARC, was performed with a starting resolution of 15 Å, followed by particle extraction to bin 1. A second round of nonuniform refinement was carried out, maintaining the initial resolution of 15 Å and the dynamic micelle mask. This was followed by a local refinement with an initial resolution of 9 Å, using a static mask for TM helices. A 3D classification was then performed with an initial resolution of 15 Å, target resolution of 2.5 Å and a focused mask for TM helices to further clean up the subset of particles. Last, a nonuniform refinement was applied to the selected volume, using an initial resolution of 9 Å, a mask for the TM helices, and minimizing over per-particle scale at each iteration of refinement. Map sharpening was applied using a B-factor estimated from the Guinier Plot, and the nominal resolution was determined according the gold-standard Fourier shell correlation (FSC) criterion at 0.143 (Table 1).

In the case of choline-bound sfCHT, two rounds of heterogeneous, nonuniform and local refinement were conducted. The heterogeneous and nonuniform refinements were performed with an initial resolution of 15 Å and a dynamic mask including the micelle, whereas the local refinement was carried out with an initial resolution of 9 Å and a static mask focused on the TM helices. Subsequently, particles were extracted to bin 1, and nonuniform and local refinements were performed again with the same parameters, incorporating the minimization over per-particle scale at each iteration of refinement. Map sharpening was applied using a B-factor estimated from the Guinier Plot, and the nominal resolution was determined according the gold-standard FSC criterion at 0.143 (Table 1).

This workflow was optimized after extensive testing to address the challenges of this dataset, which is particularly difficult due to the small particle size and the predominance of TM helices within the micelle. While we tested common cryo-EM processing tools such as CTF refinement and reference-based motion correction, these did not improve the reconstruction. The described approach proved to be the most effective for this sample. We also explored the conformational heterogeneity of the cryo-EM datasets to identify potential alternative transporter states using 3D classification without alignment and 3D variability analysis in cryoSPARC (*43*); however, these attempts did not yield additional conformations.

### Model building

In the case of model building of Na^+^-bound sfCHT, we generated an initial model using ModelAngelo (*44*). After rigid-body fitting into our 2.83-Å-resolution cryo-EM map in UCSF ChimeraX (*45*), a Na^+^ ion was manually added using Coot (*46*). Residues from 1 to 21, from 70 to 80, and the C-terminal 7 residues could not be assigned to any cryo-EM density, so were removed from the model. A final real-space refinement in Phenix was performed to improve the fit and to optimize stereochemistry (*47*). For model building of choline-bound sfCHT, the Na^+^-bound sfCHT model was rigid-body fitted into our 3.28-Å-resolution cryo-EM map in UCSF ChimeraX (*45*). Na^+^, H_2_O, and choline molecules were subsequently added using Coot (*46*). Following this, a final real-space refinement was carried out using Phenix to obtain an optimal geometry model (*47*). Figures were prepared with UCSF ChimeraX (*45*), and the tunnel-radius analysis was performed using HOLE (*48*).

### Reconstitution into PLs

WT and variant sfCHT were reconstituted in *E. coli* polar lipids (Avanti Lipids), as previously described (*49*). In brief, lipids were dried under N_2_ and suspended in reconstitution buffer containing 20 mM tris-HCl (pH 7.4), 150 mM KCl. The suspension was then sonicated to clarity, and purified sfCHT was added to a 1:50 protein/lipid ratio (w/w). Then, liposomes were destabilized by the addition of 1.25% β-d-octylglucoside (OG) and incubated on ice with occasional agitation for 5 min. DDM and OG were removed by dialysis for 40 hours at 4°C against 100 volumes of dialysis buffer [20 mM tris (pH 7.4), 150 mM KCl]. PL suspensions were frozen in liquid N_2_ and stored at −80°C until use.

### Choline transport assays in PLs

Choline uptake assays were initiated by mixing 20 μl of ice-cold PLs with 180 μl of transport buffer containing 10 mM HEPES (pH 7.4), 2 mM CaCl_2_, and 1 mM MgSO_4_. To assess Na^+^ dependence, the buffer was supplemented with either 137 mM NaCl (Na^+^-containing conditions) or 137 mM KCl (Na^+^-free conditions), together with 5 mM KCl. In experiments evaluating the role of potassium, 137 mM NMDG was used in place of KCl. The pH of Na^+^-free buffers was adjusted with 10 N KOH, whereas NMDG-containing buffers were adjusted with 5 N HCl. Transport reactions were performed in the presence of [^3^H]-choline chloride (1 μCi/ml; PerkinElmer, Waltham, MA, USA) and unlabeled choline chloride added to the desired final concentration.

Transport assays were carried out at room temperature for the indicated time periods and terminated by the addition of 2 ml of ice-cold stop buffer (5 mM choline chloride in transport buffer), followed by rapid filtration through 0.45-μm pore-size membrane filters (Sartorius Stedim Biotech). Filters were washed twice with 2 ml of cold stop buffer, dried, and the retained radioactivity was quantified by scintillation counting. Choline uptake was normalized to the sfCHT protein content of each PL preparation. Data are reported as mean ± SD from three independent experiments performed on different days using separate reconstitutions.

### sfCHT orientation in PLs

Liposomes reconstituted with His-tagged sfCHT were treated with HRV-3C protease (2 hours, at 4°C), adding 1 mM dithiothreitol and 0.5 mM EDTA to the buffer. Addition of 1% DDM allows for the complete digestion of the His tag. Then, proteins were separated by SDS-PAGE on 10% polyacrylamide gels and transferred to Immobilon-P membranes (Millipore, Billerica, MA). After incubation with anti-HA high-affinity antibody (Thermo Fisher Scientific, 1:2000), proteins were detected using a horseradish peroxidase–conjugated secondary antibody and an enhanced chemiluminescence detection kit (GE Healthcare). Blots were analyzed densitometrically using ImageJ software (*50*). N-His–tagged HRV-3C protease was obtained from the Institute for Research in Biomedicine Barcelona Protein Expression Core Facility.

### PELE

PELE is a MC molecular modeling software capable of mapping complex intermolecular biophysical problems, such as ligand migration, binding site search, local induced fit, etc. (*23*). At each MC step PELE performs multiple sampling routines including: (i) ligand random translation and rotation, (ii) protein backbone motion along a randomly chosen normal mode, (iii) side-chain sampling of all amino acids within 6 Å from the ligand, and (iv) overall minimization of the whole system. PELE global exploration (*51*) (also known as SiteFinder) was first used as an unsupervised docking of choline from outside of the transporter to explore different poses. The protocol involved 255 computing cores for 24 hours, where each core performed a (search) trajectory that was randomly placed at the surface of the transporter, for a total sampling of 200,000 PELE MC steps. We found a minimum energy pose at the intracellular tunnel exit (Fig. 3B). From this position, a series of successive local induced fit simulations were run to identify potential inner binding poses along the intracellular tunnel and within the substrate-binding site. Each induced fit simulation was allowed to explore a 9-Å sphere around the initial ligand position, involving 128 computing cores for 6 hours using the Adaptive PELE protocol. This protocol consists of 49 successive shorter runs, epochs, where 50% of the cores are sent to populate newly found substrate positions, aiming at completing the energy landscape. In total, the induced fit refinement resulted in half a million PELE MC steps. Simulations used the 2005 all atom optimized potentials for liquid simulations (OPLS) force field, with an explicit variable dielectric surface generalized born (SGB) solvent (*52*). Anisotropic network modeling was included to sample the protein backbone, using the lowest 6 normal modes, using default PELE parameters (*23*).

### Molecular dynamics simulations

The all-atom membrane system was generated using the CHARMM-GUI Membrane Builder web server, following the standard workflow and using the cryo-EM–derived structure with a bound choline residue positioned at the pore entrance (*53*, *54*). The system was prepared for production simulations using the standard CHARMM-GUI staged minimization and equilibration protocol, in which positional restraints are progressively released (*55*, *56*). All simulations used the CHARMM36 additive force field for proteins and lipids, with CHARMM-compatible parameters for the choline residue (*57*, *58*) and a TIP3P water model. Long-range electrostatic interactions were treated using the particle mesh Ewald method, with real-space cutoffs and nonbonded interactions settings consistent with CHARMM-GUI defaults. Simulations were carried out under periodic boundary conditions in the NPT ensemble using GROMACS (*59*). The temperature was maintained at 303.15 K using the Nosé-Hoover thermostat, with separate coupling groups for protein, lipids, and solvent. Pressure was maintained at 1 bar using the stochastic velocity rescaling (C-rescale) barostat with semi-isotropic coupling, allowing independent fluctuations in the membrane plane and along the membrane normal, consistent with standard membrane simulation protocols.

Twenty independent unbiased replicas were simulated for 100 ns each, differing only in their initial velocity assignments. For simulations in which the choline residue remained within the pore region at the end of 100 ns (a total of 6), trajectories were extended to simulation times exceeding 2 μs to further sample pore residency and dynamics.

### Mutagenesis and transfection of CHT1 variants

HeLa cells were maintained at 37°C in a humidified 5% CO_2_ environment in Dulbecco’s modified Eagle’s medium supplemented with 10% fetal bovine serum, penicillin (50 U/ml), streptomycin (50 μg/ml), and 2 mM l-glutamine. HeLa cells were transiently transfected in a 24-well plate with 400 ng per well with the endocytosis-resistant human CHT1-LV/AA variant (*60*) (provided by R. Blakely, Florida Atlantic University, USA) or CHT1 variants using Lipofectamine 2000 (Invitrogen, Carlsbad, USA). Single-point substitutions were introduced using the QuikChange mutagenesis kit (Stratagene, San Diego, USA). All substitutions were verified by sequencing. Choline transport assays were carried out 24 hours after transfection. Endocytosis-resistant human CHT1-LV/AA variant, we refer as WT CHT1 in the context of this study, was used as template for the generation of CHT1 variants.

### Visualization of CHT1 variants by fluorescence microscopy

To analyze the effect of the substitutions on CHT1 protein expression and plasma membrane localization, fluorescence microscopy of WT and mutant transporters was performed on a semiconfluent monolayer of transfected HeLa cells cultured on glass coverslips. Glass coverslip-grown cells were rinsed three times with phosphate-buffered saline–Ca^2+^–Mg^2+^ and fixed for 5 min in ice-cold methanol. Fixed cells were blocked and permeabilized in blocking buffer (4% fetal bovine serum and 0.5% Triton X-100 in PBS) for 1 hour and then incubated for 1 hour with primary antibody (anti-CHT, #CHT-Go-Af890, Nittobo Medical). Secondary donkey–anti-goat–Alexa 546 antibody (Life Technologies) was incubated for 2 hours protected from light and rinsed three times with phosphate-buffered saline. Nuclear staining was performed by incubating Hoechst (1 μg/ml; Thermo Fisher Scientific) for 10 min, rinsed three times with phosphate-buffered saline and then mounted with aqua-poly/mount coverslipping medium (Polysciences Inc.). Images were taken using a Nikon E1000 upright epifluorescence microscope. All images were captured during 200 ms.

### Choline transport assays in HeLa cells

Choline uptake was measured in WT and variant CHT1, and mock-transfected HeLa cells, as previously described (*24*), by exposing replicate cultures at room temperature to [^3^H]-labeled choline chloride (1 μCi/ml; PerkinElmer, Waltham, USA) in transport buffer [10 mM HEPES (pH 7.4), 137 mM NaCl, 5 mM KCl, 2 mM CaCl_2_, and 1 mM MgSO_4_]. Initial rates of transport were determined using an incubation period of 2 min, as described (*60*). Assays were terminated by washing with an excess volume of ice-cold stop buffer (5 mM choline chloride in transport buffer). Transporter-mediated choline uptake was calculated by subtracting the uptake measured in mock-transfected cells.

For saturation kinetics, transfected cells were incubated with [^3^H] choline chloride (1 μCi/ml) and varying concentrations of unlabeled choline chloride (0 to 100 or 100 to 500 μM, depending on the mutant to be analyzed). Cold substrates were prepared at 100 mM, aliquoted, and stored at −20°C until use. Aliquots were thawed only once to reduce variability. Each replicate of the kinetic studies was performed simultaneously for WT and variant CHT1. The Michaelis-Menten and Eadie-Hofstee equations were then applied, and the kinetic parameters derived from this method were confirmed by linear regression analysis of the derived Eadie-Hofstee plots using the GraphPad Prism software. Data are expressed as the mean ± SD of three experiments performed on different days and batches of cells.

## References

[1] M. J. Wargo, Homeostasis and catabolism of choline and glycine betaine: Lessons from Pseudomonas aeruginosa. Appl. Environ. Microbiol. 79, 2112–2120 (2013). 23354714 10.1128/AEM.03565-12 PMC3623244

[2] S. H. Zeisel, Choline: Essential for brain development and function. Adv. Pediatr. 44, 263–295 (1997). 9265973

[3] S. H. Zeisel, Choline: Critical role during fetal development and dietary requirements in adults. Annu. Rev. Nutr. 26, 229–250 (2006). 16848706 10.1146/annurev.nutr.26.061505.111156 PMC2441939

[4] O. Geiger, I. M. Lopez-Lara, C. Sohlenkamp, Phosphatidylcholine biosynthesis and function in bacteria. Biochim. Biophys. Acta 1831, 503–513 (2013). 22922101 10.1016/j.bbalip.2012.08.009

[5] C. Ziegler, E. Bremer, R. Kramer, The BCCT family of carriers: From physiology to crystal structure. Mol. Microbiol. 78, 13–34 (2010). 20923416 10.1111/j.1365-2958.2010.07332.x

[6] M. A. Salvano, T. A. Lisa, C. E. Domenech, Choline transport in Pseudomonas aeruginosa. Mol. Cell. Biochem. 85, 81–89 (1989). 2498639 10.1007/BF00223517

[7] M. J. Wargo, T. C. Ho, M. J. Gross, L. A. Whittaker, D. A. Hogan, GbdR regulates Pseudomonas aeruginosa plcH and pchP transcription in response to choline catabolites. Infect. Immun. 77, 1103–1111 (2009). 19103776 10.1128/IAI.01008-08 PMC2643652

[8] O. A. Ojiakor, R. J. Rylett, Modulation of sodium-coupled choline transporter CHT function in health and disease. Neurochem. Int. 140, 104810 (2020). 32768485 10.1016/j.neuint.2020.104810

[9] S. Apparsundaram, S. M. Ferguson, A. L. George Jr., R. D. Blakely, Molecular cloning of a human, hemicholinium-3-sensitive choline transporter. Biochem. Biophys. Res. Commun. 276, 862–867 (2000). 11027560 10.1006/bbrc.2000.3561

[10] T. Okuda, T. Haga, Functional characterization of the human high-affinity choline transporter. FEBS Lett. 484, 92–97 (2000). 11068039 10.1016/s0014-5793(00)02134-7

[11] H. Iwamoto, R. D. Blakely, L. J. De Felice, Na^+^, Cl^–^, and pH dependence of the human choline transporter (hCHT) in Xenopus oocytes: The proton inactivation hypothesis of hCHT in synaptic vesicles. J. Neurosci. 26, 9851–9859 (2006). 17005849 10.1523/JNEUROSCI.1862-06.2006 PMC6674471

[12] Y. Qiu, Y. Gao, B. Huang, Q. Bai, Y. Zhao, Transport mechanism of presynaptic high-affinity choline uptake by CHT1. Nat. Struct. Mol. Biol. 31, 701–709 (2024). 38589607 10.1038/s41594-024-01259-w

[13] J. Xue, H. Chen, Y. Wang, Y. Jiang, Structural mechanisms of human sodium-coupled high-affinity choline transporter CHT1. Cell Discov. 10, 116 (2024). 39587078 10.1038/s41421-024-00731-7 PMC11589582

[14] Y. Qiu, Y. Gao, Q. Bai, Y. Zhao, Ion coupling and inhibitory mechanisms of the human presynaptic high-affinity choline transporter CHT1. Structure 31, 701–709 (2024). 10.1016/j.str.2024.11.009 39657660

[15] C. Oswald, S. H. Smits, M. Hoing, L. Sohn-Bosser, L. Dupont, D. Le Rudulier, L. Schmitt, E. Bremer, Crystal structures of the choline/acetylcholine substrate-binding protein ChoX from Sinorhizobium meliloti in the liganded and unliganded-closed states. J. Biol. Chem. 283, 32848–32859 (2008). 18779321 10.1074/jbc.M806021200

[16] C. Perez, C. Koshy, S. Ressl, S. Nicklisch, R. Kramer, C. Ziegler, Substrate specificity and ion coupling in the Na^+^/betaine symporter BetP. EMBO J. 30, 1221–1229 (2011). 21364531 10.1038/emboj.2011.46 PMC3094121

[17] Y. Du, W. W. Shi, Y. X. He, Y. H. Yang, C. Z. Zhou, Y. Chen, Structures of the substrate-binding protein provide insights into the multiple compatible solute binding specificities of the Bacillus subtilis ABC transporter OpuC. Biochem. J. 436, 283–289 (2011). 21366542 10.1042/BJ20102097

[18] M. Pittelkow, B. Tschapek, S. H. Smits, L. Schmitt, E. Bremer, The crystal structure of the substrate-binding protein OpuBC from Bacillus subtilis in complex with choline. J. Mol. Biol. 411, 53–67 (2011). 21658392 10.1016/j.jmb.2011.05.037

[19] N. Barland, A. S. Rueff, G. Cebrero, C. A. J. Hutter, M. A. Seeger, J. W. Veening, C. Perez, Mechanistic basis of choline import involved in teichoic acids and lipopolysaccharide modification. Sci. Adv. 8, eabm1122 (2022). 35235350 10.1126/sciadv.abm1122 PMC8890701

[20] T. Yang, Y. Nian, H. Lin, J. Li, X. Lin, T. Li, R. Wang, L. Wang, G. A. Beattie, J. Zhang, M. Fan, Structure and mechanism of the osmoregulated choline transporter BetT. Sci. Adv. 10, eado6229 (2024). 39141726 10.1126/sciadv.ado6229 PMC11323884

[21] M. Knipper, I. Boekhoff, H. Breer, Isolation and reconstitution of the high-affinity choline carrier. FEBS Lett. 245, 235–237 (1989). 2924923 10.1016/0014-5793(89)80228-5

[22] Y. Shi, Common folds and transport mechanisms of secondary active transporters. Annu. Rev. Biophys. 42, 51–72 (2013). 23654302 10.1146/annurev-biophys-083012-130429

[23] D. Lecina, J. F. Gilabert, V. Guallar, Adaptive simulations, towards interactive protein-ligand modeling. Sci. Rep. 7, 8466 (2017). 28814780 10.1038/s41598-017-08445-5 PMC5559483

[24] J. Rullo-Tubau, M. Martinez-Molledo, P. Bartoccioni, I. Puch-Giner, A. Arias, S. Saen-Oon, C. Stephan-Otto Attolini, R. Artuch, L. Diaz, V. Guallar, E. Errasti-Murugarren, M. Palacin, O. Llorca, Structure and mechanisms of transport of human Asc1/CD98hc amino acid transporter. Nat. Commun. 15, 2986 (2024). 38582862 10.1038/s41467-024-47385-3 PMC10998858

[25] C. F. Rodriguez, P. Escudero-Bravo, L. Diaz, P. Bartoccioni, C. Garcia-Martin, J. G. Gilabert, J. Boskovic, V. Guallar, E. Errasti-Murugarren, O. Llorca, M. Palacin, Structural basis for substrate specificity of heteromeric transporters of neutral amino acids. Proc. Natl. Acad. Sci. U.S.A. 118, e2113573118 (2021). 34848541 10.1073/pnas.2113573118 PMC8670485

[26] E. Errasti-Murugarren, J. Fort, P. Bartoccioni, L. Diaz, E. Pardon, X. Carpena, M. Espino-Guarch, A. Zorzano, C. Ziegler, J. Steyaert, J. Fernandez-Recio, I. Fita, M. Palacin, L amino acid transporter structure and molecular bases for the asymmetry of substrate interaction. Nat. Commun. 10, 1807 (2019). 31000719 10.1038/s41467-019-09837-z PMC6472337

[27] I. Tascon, J. S. Sousa, R. A. Corey, D. J. Mills, D. Griwatz, N. Aumuller, V. Mikusevic, P. J. Stansfeld, J. Vonck, I. Hanelt, Structural basis of proton-coupled potassium transport in the KUP family. Nat. Commun. 11, 626 (2020). 32005818 10.1038/s41467-020-14441-7 PMC6994465

[28] S. Weyand, T. Shimamura, O. Beckstein, M. S. Sansom, S. Iwata, P. J. Henderson, A. D. Cameron, The alternating access mechanism of transport as observed in the sodium-hydantoin transporter Mhp1. J. Synchrotron Radiat. 18, 20–23 (2011). 21169684 10.1107/S0909049510032449 PMC3004247

[29] O. Jardetzky, Protein dynamics and conformational transitions in allosteric proteins. Prog. Biophys. Mol. Biol. 65, 171–219 (1996). 9062432 10.1016/s0079-6107(96)00010-7

[30] M. Hiraizumi, T. Akashi, K. Murasaki, H. Kishida, T. Kumanomidou, N. Torimoto, O. Nureki, I. Miyaguchi, Transport and inhibition mechanism of the human SGLT2-MAP17 glucose transporter. Nat. Struct. Mol. Biol. 31, 159–169 (2023). 38057552 10.1038/s41594-023-01134-0 PMC10803289

[31] M. O. Jensen, Y. Yin, E. Tajkhorshid, K. Schulten, Sugar transport across lactose permease probed by steered molecular dynamics. Biophys. J. 93, 92–102 (2007). 17434947 10.1529/biophysj.107.103994 PMC1914442

[32] S. Skovstrup, L. David, O. Taboureau, F. S. Jorgensen, A steered molecular dynamics study of binding and translocation processes in the GABA transporter. PLOS ONE 7, e39360 (2012). 22737235 10.1371/journal.pone.0039360 PMC3380839

[33] Y. Wei, R. Li, Y. Meng, T. Hu, J. Zhao, Y. Gao, Q. Bai, N. Li, Y. Zhao, Transport mechanism and pharmacology of the human GlyT1. Cell 187, 1719–1732.e14 (2024). 38513663 10.1016/j.cell.2024.02.026

[34] J. H. Yoon, S. J. Kang, K. H. Oh, T. K. Oh, Salimicrobium flavidum sp. nov., isolated from a marine solar saltern. Int. J. Syst. Evol. Microbiol. 59, 2839–2842 (2009). 19628596 10.1099/ijs.0.010215-0

[35] D. Van Thuoc, T. T. Loan, N. T. Tra, Accumulation of ectoines by halophilic bacteria isolated from fermented shrimp paste: An adaptation mechanism to salinity, temperature, and pH stress. Curr. Microbiol. 78, 2355–2366 (2021). 33830319 10.1007/s00284-021-02481-1

[36] R. J. Cater, D. Mukherjee, E. Gil-Iturbe, S. K. Erramilli, T. Chen, K. Koo, N. Santander, A. Reckers, B. Kloss, T. Gawda, B. C. Choy, Z. Zhang, A. Katewa, A. Larpthaveesarp, E. J. Huang, S. W. J. Mooney, O. B. Clarke, S. W. Yee, K. M. Giacomini, A. A. Kossiakoff, M. Quick, T. Arnold, F. Mancia, Structural and molecular basis of choline uptake into the brain by FLVCR2. Nature 629, 704–709 (2024). 38693257 10.1038/s41586-024-07326-y PMC11168207

[37] K. Ri, T. H. Weng, A. Claveras Cabezudo, W. Josting, Y. Zhang, A. Bazzone, N. C. P. Leong, S. Welsch, R. T. Doty, G. Gursu, T. J. Y. Lim, S. L. Schmidt, J. L. Abkowitz, G. Hummer, D. Wu, L. N. Nguyen, S. Safarian, Molecular mechanism of choline and ethanolamine transport in humans. Nature 630, 501–508 (2024). 38778100 10.1038/s41586-024-07444-7 PMC11168923

[38] Y. Son, T. C. Kenny, A. Khan, K. Birsoy, R. K. Hite, Structural basis of lipid head group entry to the Kennedy pathway by FLVCR1. Nature 629, 710–716 (2024). 38693265 10.1038/s41586-024-07374-4 PMC11188936

[39] J. Pei, B. H. Kim, N. V. Grishin, PROMALS3D: A tool for multiple protein sequence and structure alignments. Nucleic Acids Res. 36, 2295–2300 (2008). 18287115 10.1093/nar/gkn072 PMC2367709

[40] A. M. Waterhouse, J. B. Procter, D. M. Martin, M. Clamp, G. J. Barton, Jalview Version 2—A multiple sequence alignment editor and analysis workbench. Bioinformatics 25, 1189–1191 (2009). 19151095 10.1093/bioinformatics/btp033 PMC2672624

[41] A. Punjani, J. L. Rubinstein, D. J. Fleet, M. A. Brubaker, cryoSPARC: Algorithms for rapid unsupervised cryo-EM structure determination. Nat. Methods 14, 290–296 (2017). 28165473 10.1038/nmeth.4169

[42] T. Bepler, A. Morin, M. Rapp, J. Brasch, L. Shapiro, A. J. Noble, B. Berger, Positive-unlabeled convolutional neural networks for particle picking in cryo-electron micrographs. Nat. Methods 16, 1153–1160 (2019). 31591578 10.1038/s41592-019-0575-8 PMC6858545

[43] A. Punjani, D. J. Fleet, 3D variability analysis: Resolving continuous flexibility and discrete heterogeneity from single particle cryo-EM. J. Struct. Biol. 213, 107702 (2021). 33582281 10.1016/j.jsb.2021.107702

[44] K. Jamali, L. Kall, R. Zhang, A. Brown, D. Kimanius, S. H. W. Scheres, Automated model building and protein identification in cryo-EM maps. Nature 628, 450–457 (2024). 38408488 10.1038/s41586-024-07215-4 PMC11006616

[45] T. D. Goddard, C. C. Huang, E. C. Meng, E. F. Pettersen, G. S. Couch, J. H. Morris, T. E. Ferrin, UCSF ChimeraX: Meeting modern challenges in visualization and analysis. Protein Sci. 27, 14–25 (2018). 28710774 10.1002/pro.3235 PMC5734306

[46] P. Emsley, K. Cowtan, Coot: Model-building tools for molecular graphics. Acta Crystallogr. D Biol. Crystallogr. 60, 2126–2132 (2004). 15572765 10.1107/S0907444904019158

[47] P. D. Adams, P. V. Afonine, G. Bunkoczi, V. B. Chen, I. W. Davis, N. Echols, J. J. Headd, L. W. Hung, G. J. Kapral, R. W. Grosse-Kunstleve, A. J. McCoy, N. W. Moriarty, R. Oeffner, R. J. Read, D. C. Richardson, J. S. Richardson, T. C. Terwilliger, P. H. Zwart, PHENIX: A comprehensive Python-based system for macromolecular structure solution. Acta Crystallogr. D Biol. Crystallogr. 66, 213–221 (2010). 20124702 10.1107/S0907444909052925 PMC2815670

[48] O. S. Smart, J. G. Neduvelil, X. Wang, B. A. Wallace, M. S. Sansom, HOLE: A program for the analysis of the pore dimensions of ion channel structural models. J. Mol. Graph. 14, 354–360 (1996). 9195488 10.1016/s0263-7855(97)00009-x

[49] L. Kowalczyk, M. Ratera, A. Paladino, P. Bartoccioni, E. Errasti-Murugarren, E. Valencia, G. Portella, S. Bial, A. Zorzano, I. Fita, M. Orozco, X. Carpena, J. L. Vazquez-Ibar, M. Palacin, Molecular basis of substrate-induced permeation by an amino acid antiporter. Proc. Natl. Acad. Sci. U.S.A. 108, 3935–3940 (2011). 21368142 10.1073/pnas.1018081108 PMC3054010

[50] C. A. Schneider, W. S. Rasband, K. W. Eliceiri, NIH Image to ImageJ: 25 years of image analysis. Nat. Methods 9, 671–675 (2012). 22930834 10.1038/nmeth.2089 PMC5554542

[51] S. Acebes, E. Fernandez-Fueyo, E. Monza, M. F. Lucas, D. Almendral, F. J. Ruiz-Dueñas, H. Lund, A. T. Martinez, V. Guallar, Rational enzyme engineering through biophysical and biochemical modeling. ACS Catal. 6, 1624–1629 (2016).

[52] J. Li, R. Abel, K. Zhu, Y. Cao, S. Zhao, R. A. Friesner, The VSGB 2.0 model: A next generation energy model for high resolution protein structure modeling. Proteins 79, 2794–2812 (2011). 21905107 10.1002/prot.23106 PMC3206729

[53] S. Jo, T. Kim, V. G. Iyer, W. Im, CHARMM-GUI: A web-based graphical user interface for CHARMM. J. Comput. Chem. 29, 1859–1865 (2008). 18351591 10.1002/jcc.20945

[54] E. L. Wu, X. Cheng, S. Jo, H. Rui, K. C. Song, E. M. Davila-Contreras, Y. Qi, J. Lee, V. Monje-Galvan, R. M. Venable, J. B. Klauda, W. Im, CHARMM-GUI Membrane Builder toward realistic biological membrane simulations. J. Comput. Chem. 35, 1997–2004 (2014). 25130509 10.1002/jcc.23702 PMC4165794

[55] S. Jo, J. B. Lim, J. B. Klauda, W. Im, CHARMM-GUI Membrane Builder for mixed bilayers and its application to yeast membranes. Biophys. J. 97, 50–58 (2009). 19580743 10.1016/j.bpj.2009.04.013 PMC2711372

[56] J. Lee, X. Cheng, J. M. Swails, M. S. Yeom, P. K. Eastman, J. A. Lemkul, S. Wei, J. Buckner, J. C. Jeong, Y. Qi, S. Jo, V. S. Pande, D. A. Case, C. L. Brooks III, A. D. MacKerell Jr., J. B. Klauda, W. Im, CHARMM-GUI input generator for NAMD, GROMACS, AMBER, OpenMM, and CHARMM/OpenMM simulations using the CHARMM36 Additive Force Field. J. Chem. Theory Comput. 12, 405–413 (2016). 26631602 10.1021/acs.jctc.5b00935 PMC4712441

[57] J. Huang, A. D. MacKerell Jr., CHARMM36 all-atom additive protein force field: Validation based on comparison to NMR data. J. Comput. Chem. 34, 2135–2145 (2013). 23832629 10.1002/jcc.23354 PMC3800559

[58] J. B. Klauda, R. M. Venable, J. A. Freites, J. W. O’Connor, D. J. Tobias, C. Mondragon-Ramirez, I. Vorobyov, A. D. MacKerell Jr., R. W. Pastor, Update of the CHARMM all-atom additive force field for lipids: Validation on six lipid types. J. Phys. Chem. B 114, 7830–7843 (2010). 20496934 10.1021/jp101759q PMC2922408

[59] M. J. Abraham, T. Murtola, R. Schulz, S. Páll, J. C. Smith, B. Hess, E. Lindahl, GROMACS: High performance molecular simulations through multi-level parallelism from laptops to supercomputers. SoftwareX 1-2, 19–25 (2015).

[60] A. M. Ruggiero, J. Wright, S. M. Ferguson, M. Lewis, K. S. Emerson, H. Iwamoto, M. T. Ivy, E. C. Holmstrand, E. A. Ennis, C. D. Weaver, R. D. Blakely, Nonoisotopic assay for the presynaptic choline transporter reveals capacity for allosteric modulation of choline uptake. ACS Chem. Nerosci. 3, 767–781 (2012). 10.1021/cn3000718 PMC3474274 23077721

