# Supplementary material for: Structural insights into a conserved mechanism of choline translocation through CHT
Source: Sci Adv. 2026 May 29;12(22):eaec1241. doi: 10.1126/sciadv.aec1241 (PMC13220886; doi:10.1126/sciadv.aec1241)
Supplement: Supplementary file 1 — Figs. S1 to S17 Legend for movie S1 Legend for data S1 [file sciadv.aec1241_sm.pdf]

Science Advances
AAAS

Supplementary Materials for

Structural insights into a conserved mechanism of choline translocation through CHT

Jesus Vilchez-Garcia *et al.*

Corresponding author: Igor Tascón, igor.tascon@ehu.eus; Iban Ubarretxena-Belandia, ivan.ubarrechena@ehu.eus; Ekaitz Errasti-Murugarren, ekaitz_errasti@ub.edu

Sci. Adv. **12**, eaec1241 (2026)
DOI: 10.1126/sciadv.aec1241

The PDF file includes:

Figs. S1 to S17
Legend for movie S1
Legend for data S1

Other Supplementary Material for this manuscript includes the following:

Movie S1
Data S1

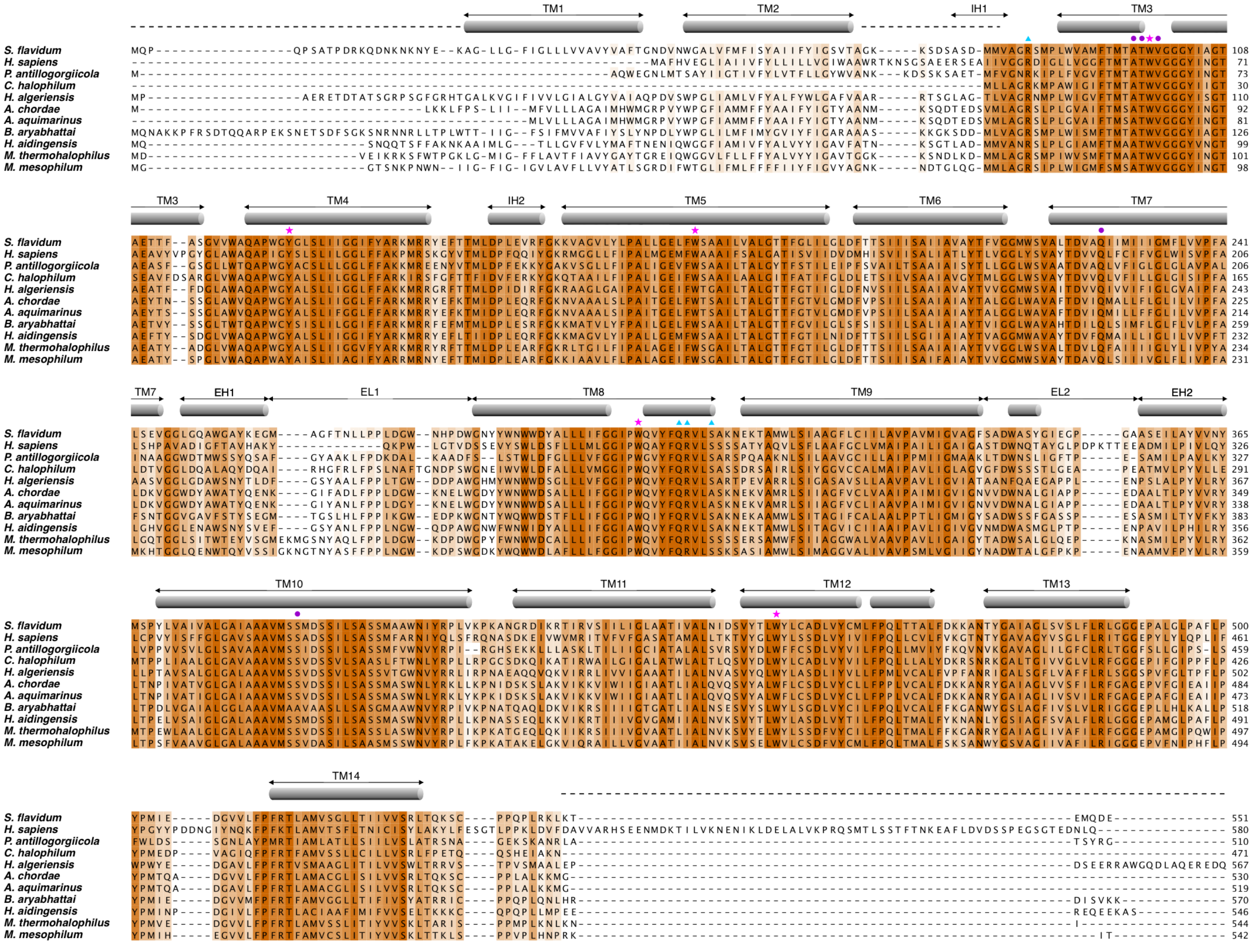

Fig. S1. Sequence alignment of SLC5A7 from *S. flavidum* with human SLC5A7 and other homologous proteins from different bacteria. Sequences of SLC5A7 from *Salimicrobium flavidum*, *Homo sapiens*, *Pseudobacteriovorax antillogorgiicola*, *Cyclobacterium halophilum*, *Halopolyspora algeriensis*, *Algoriphagus chordae*, *Algoriphagus aquimarinus*, *Bacillus aryabhattai*, *Halobacillus aidingensis*, *Melghirimyces thermohalophilus*, *Marininema mesophilum* are aligned. The sequence alignment was carried out using PROMALS3D (*39*), and the figure was prepared using Jalview (*40*). Sequence conservation is shown by coloring, with dark orange indicating full conservation. The secondary structure of sfCHT is shown with gray cylinders. Dashed lines before K22, between A69 and V81, and after L544 indicate unresolved residues in our structures due to protein flexibility in these segments. Magenta stars indicate residues constituting the substrate-binding site, purple circles residues involved in Na^+ coordination, and blue triangles indicate the identified key residues R84, Q303, R304 and S307. TM, IH, EH and EL stand for transmembrane helix, intracellular helix, extracellular helix and extracellular loop, respectively.

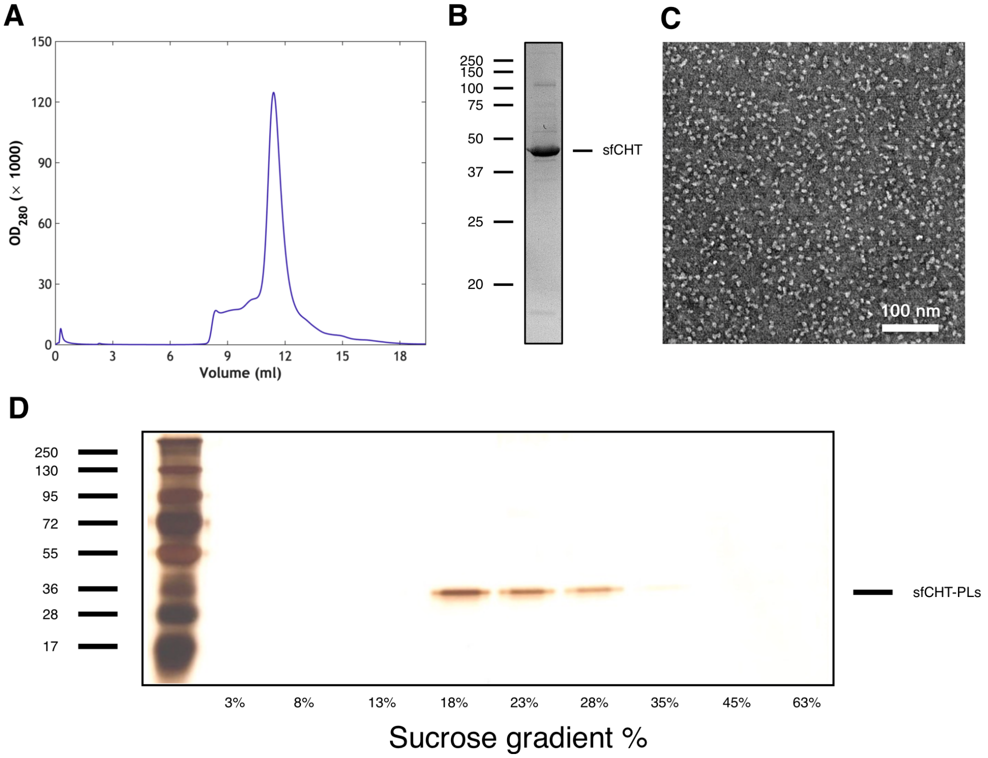

Fig. S2. sfCHT for structural biology studies. (**A**) Size exclusion chromatography profile of a representative purification of sfCHT. (**B**) SDS-PAGE showing a prominent band corresponding to purified sfCHT after size exclusion chromatography. (**C**) Representative negative staining micrograph of sfCHT illustrating a good particle distribution without protein aggregates. (**D**) Sucrose gradient of DDM-purified sfCHT and reconstituted in *E. coli* polar lipid liposomes. The layers of the gradient were analyzed by SDS-PAGE and further silver staining. Given concentrations correspond to sucrose concentrations before centrifugation.

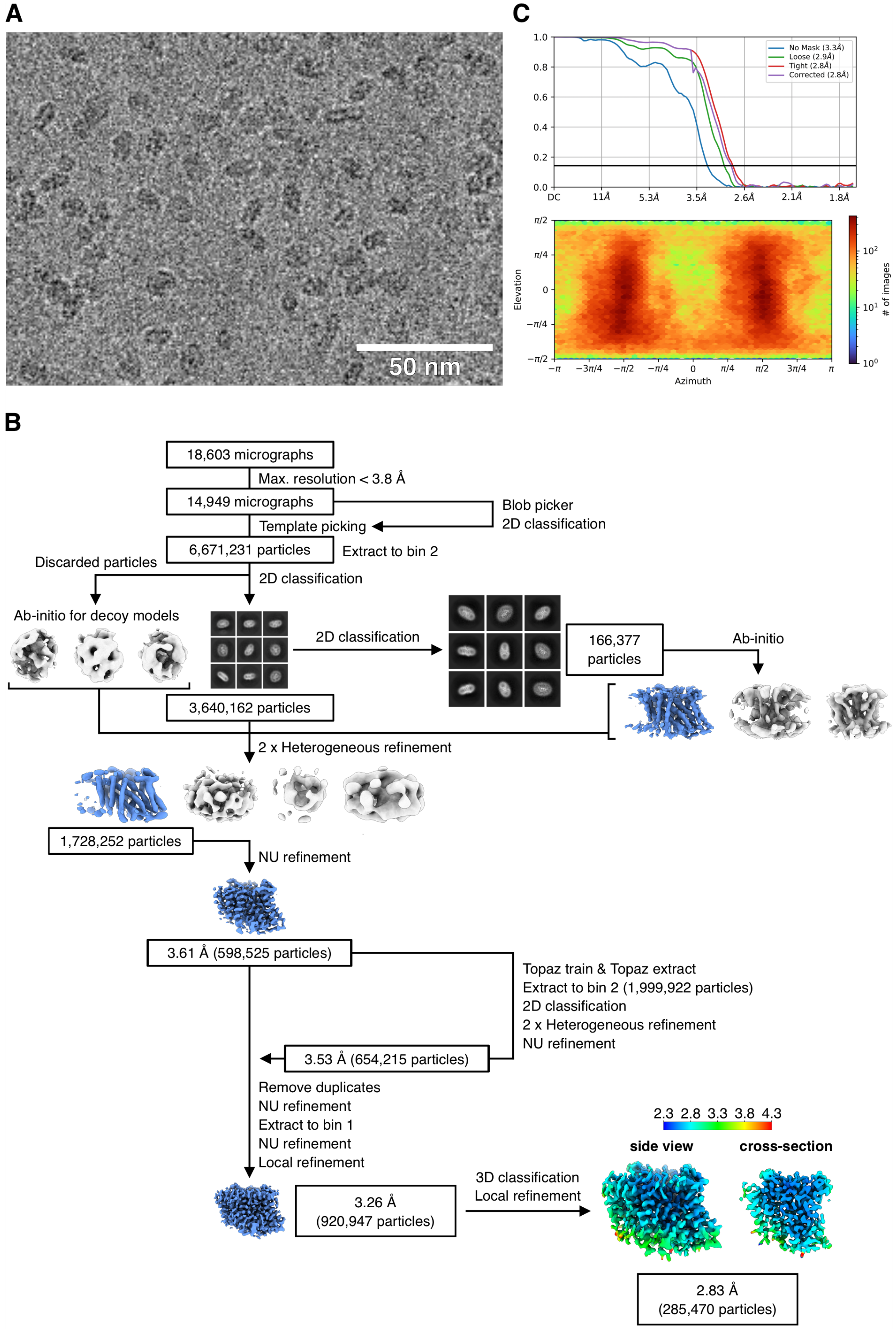

Fig. S3. Cryo-EM data processing pipeline for Na^+-bound sfCHT. (**A**) Zoomed-in view of a representative cryo-EM micrograph of Na^+-bound sfCHT. (**B**) Cryo-EM data processing workflow. (**C**) Gold-standard FSC curve (top) and Euler diagram showing particle orientation distribution (bottom).

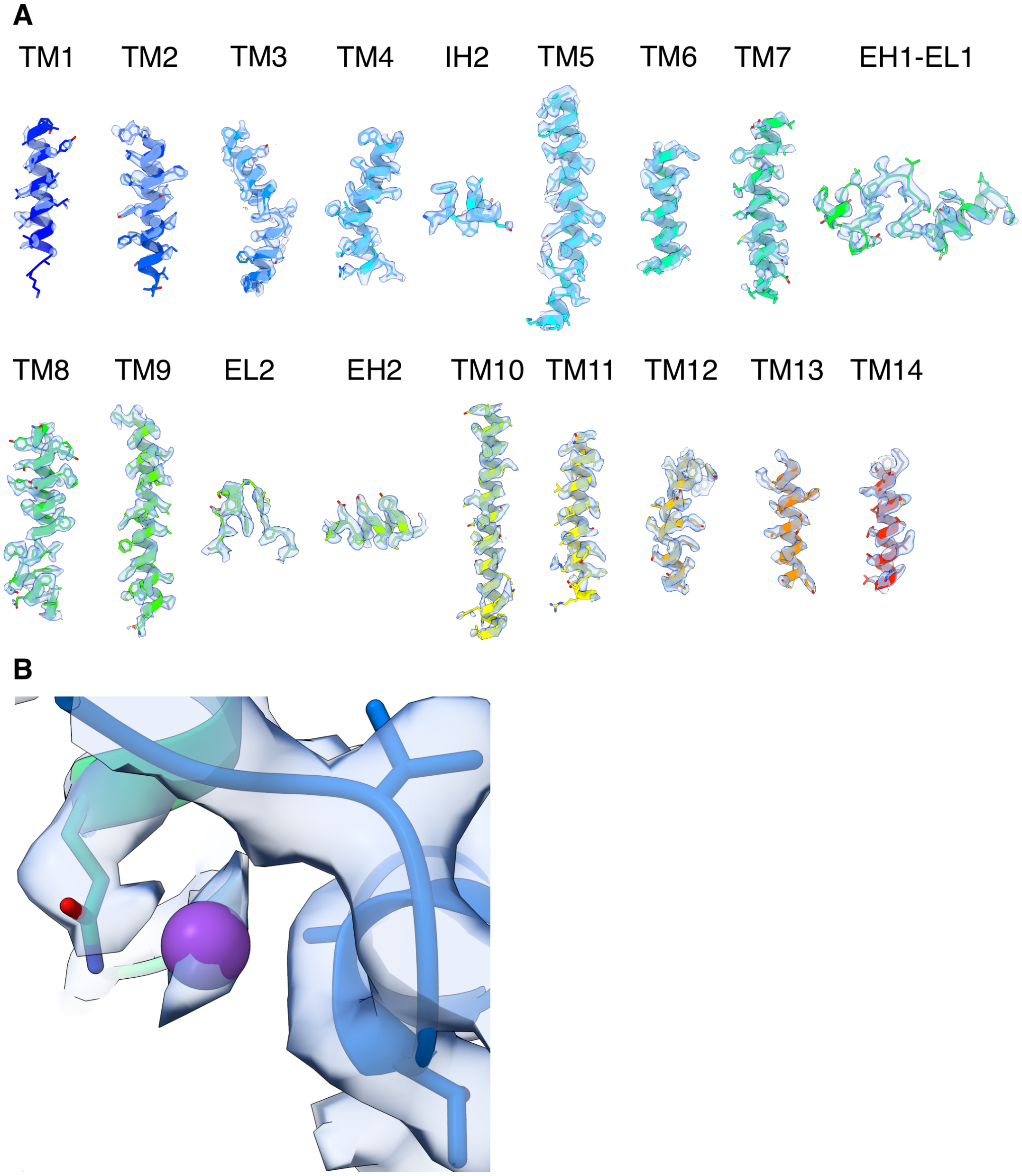

Fig. S4. Cryo-EM map and model-to-map fit of Na^+-bound sfCHT. The map threshold was set to an RMSD of 8.20 Å in all panels. (**A**) The different segments of sfCHT, named as in Supplementary Figure 1, are depicted in cartoon format with the side chains shown in sticks. The cryo-EM density map is displayed around these segments. (**B**) Close-up view of the Na^+ cation shown as spheres, interacting with residues depicted in sticks.

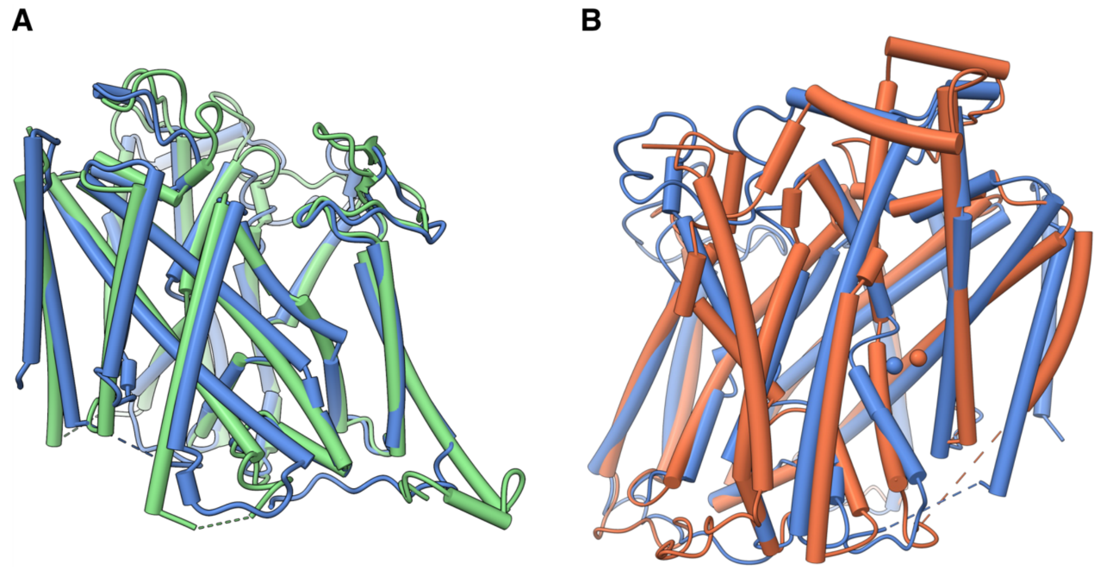

Fig. S5. Structural homology. (**A**) Side front view of superimposed Na^+-bound sfCHT (in blue) and apo CHT1 (PDB 9BFI, in green), with α-helices displayed as tubes. (**B**) Side back view of superimposed Na^+-bound sfCHT (in blue) and vSGLT (in coral), with α-helices displayed as tubes, and the Na^+ ions as spheres.

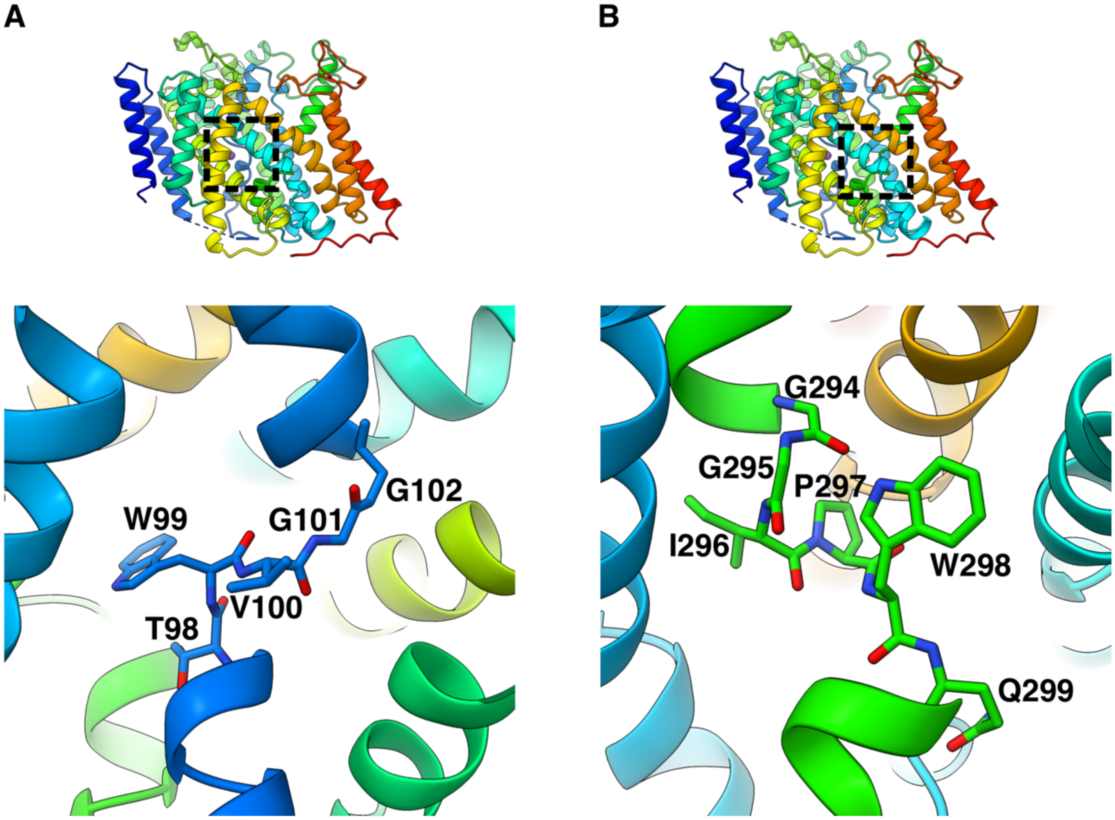

Fig. S6. Discontinuities in TM helix 3 (A) and TM helix 8 (B). The unwound segments are depicted as sticks. The upper section of each panel highlights the specific region of the protein shown in the enlarged view below.

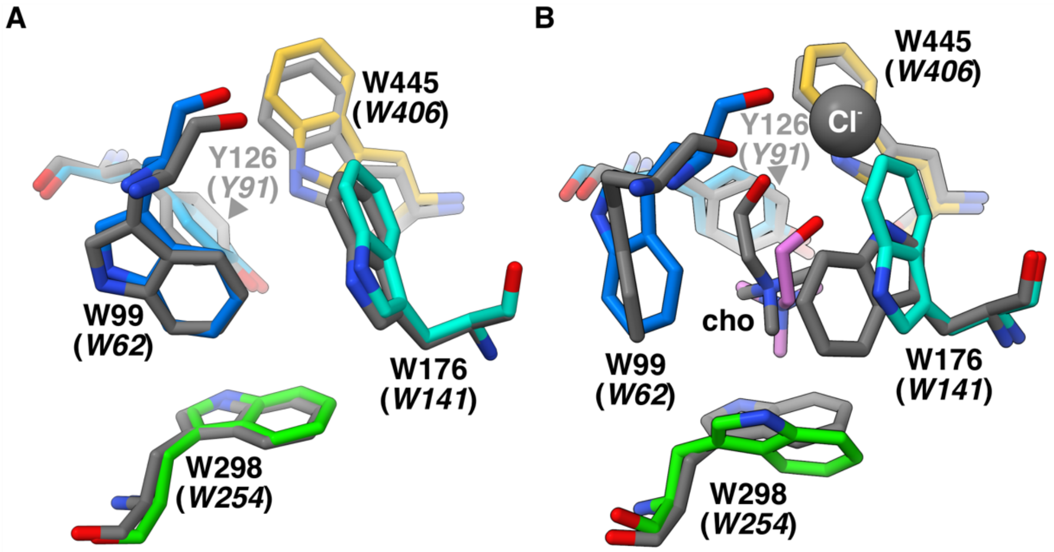

Fig. S7. Substrate-binding site comparison between sfCHT and CHT1. (**A**) Structural alignment of inward-facing Na^+-bound sfCHT (colored) and inward-open apo CHT1 (PDB: 9BFI, shown in gray). (**B**) Structural alignment of the PELE-predicted model of sfCHT with choline-bound to the substrate-binding site (colored) and the choline-bound occluded CHT1 structure (PDB: 9BFJ, shown in gray).

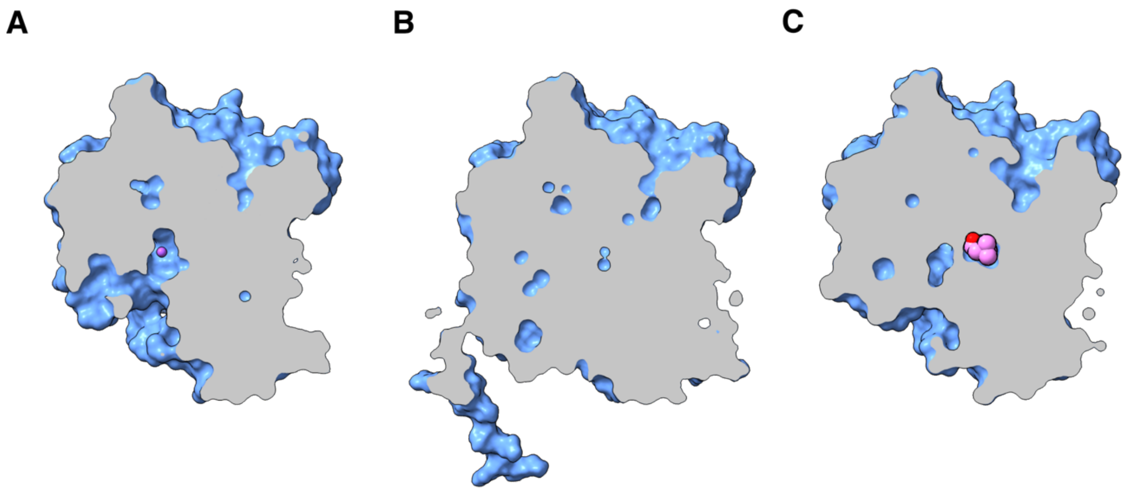

Fig. S8. Accessibility of the substrate-binding site in sfCHT. The panels illustrate a cross-section of sfCHT structures, revealing the internal architecture from a front view. The structures are represented as surface models, and the Na^+ ion is shown as a purple sphere. (**A**) In the inward-facing Na^+-bound sfCHT cryo-EM structure, a tunnel from the cation to the inner side of the membrane is observed. (**B**) The AlphaFold sfCHT model lacks any discernible intracellular tunnel. (**C**) In the PELE-derived sfCHT structure, choline is captured in an occluded state within the substrate-binding site.

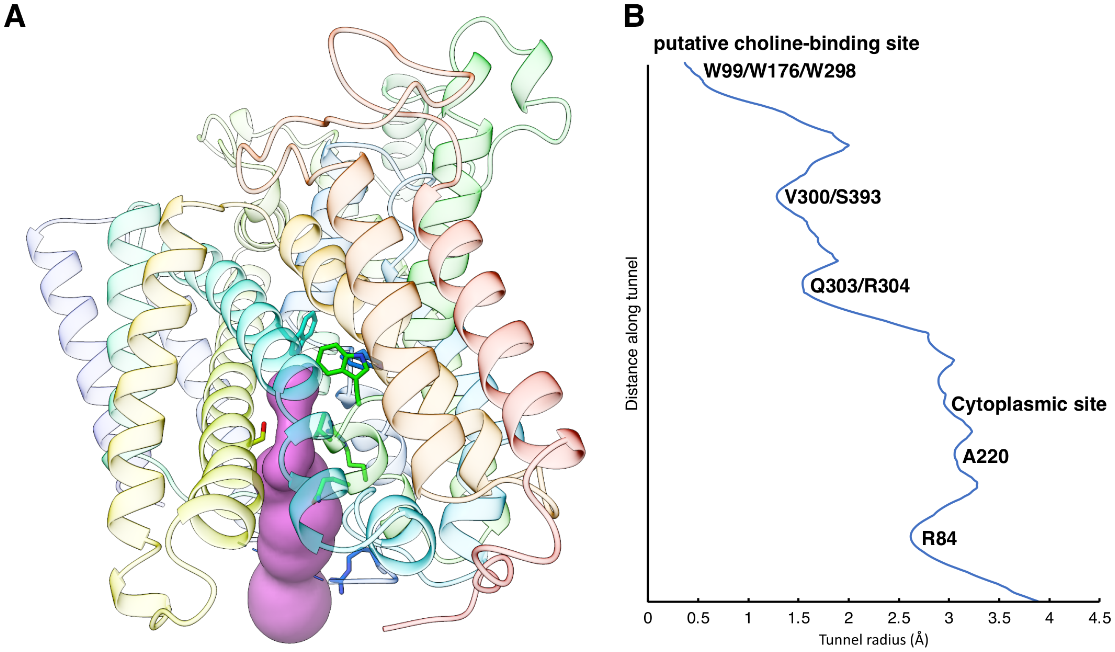

Fig. S9. **Analysis of the sfCHT intracellular tunnel radii**. (**A**) Surface representation of the intracellular tunnel in Na^+-bound sfCHT structure. The tunnel surface is colored in pink, the sfCHT atomic model is displayed as ribbons colored as in Fig. 1, and tunnel-lining residues are depicted as sticks. (**B**) Intracellular tunnel radii along the choline transition pathway in sfCHT calculated using HOLE (*47*).

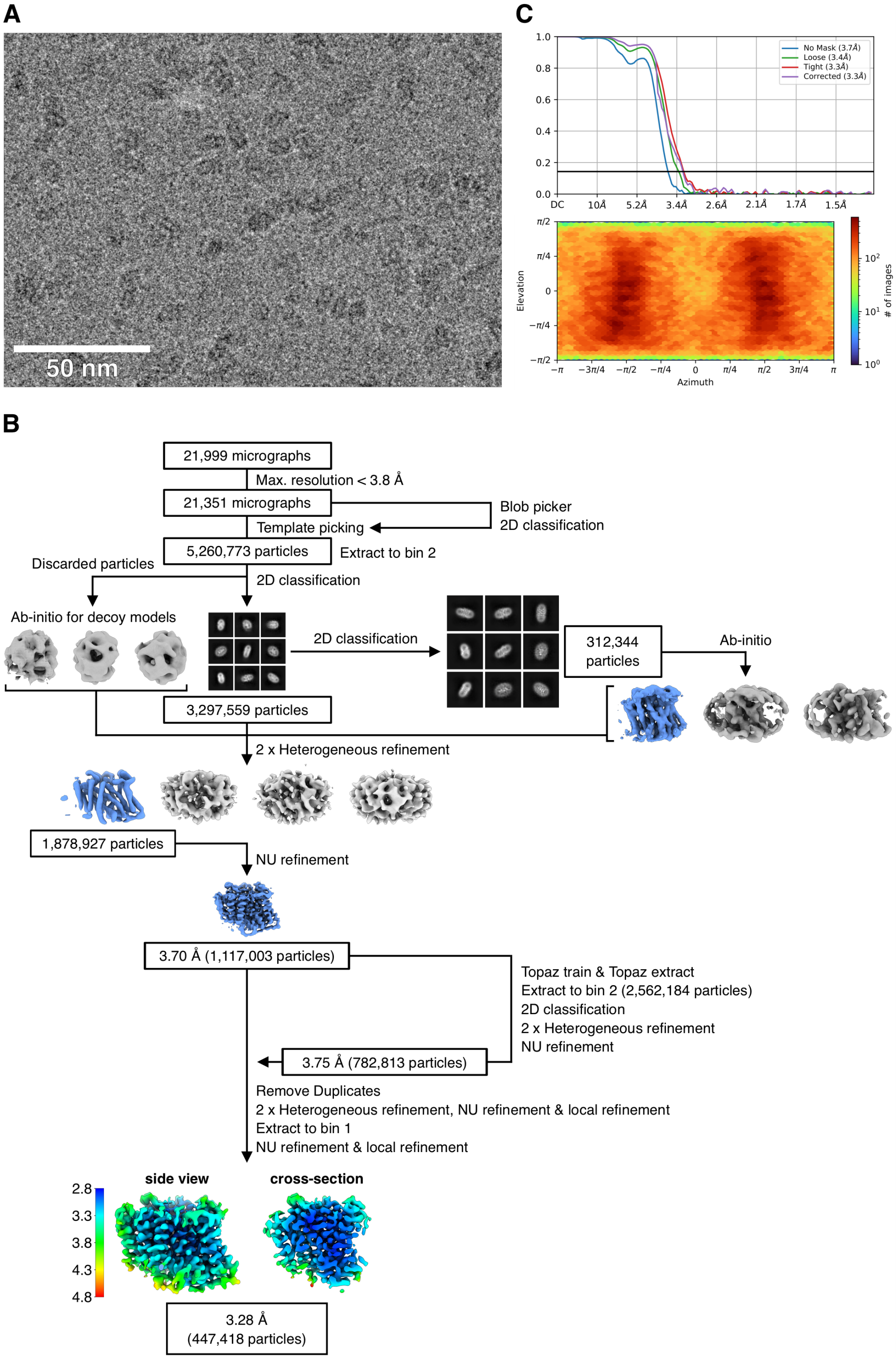

Fig. S10. Cryo-EM data processing pipeline for choline-bound sfCHT. (**A**) Zoomed-in view of a representative cryo-EM micrograph of choline-bound sfCHT. (**B**) Cryo-EM data processing workflow. (**C**) Gold-standard FSC curve (top) and Euler diagram showing particle orientation distribution (bottom).

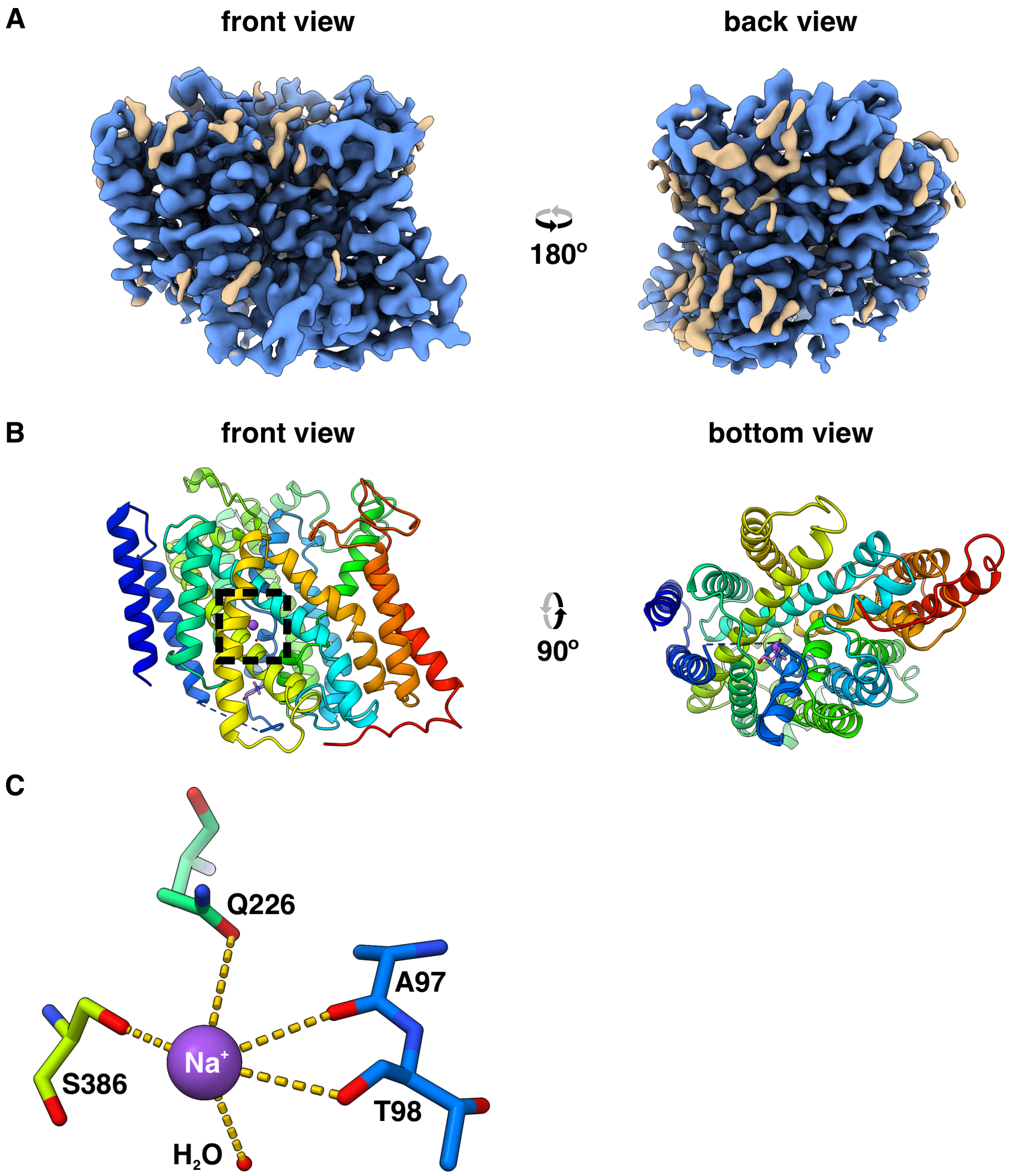

Fig. S11. Overview of the structure of choline-bound sfCHT. (**A**) Cryo-EM map of sfCHT at 3.28 Å nominal resolution viewed from the front and back side. The map regions colored in blue correspond to protein and ligand-assigned densities, whereas non-protein unassigned densities are brown colored. (**B**) Ribbon representation of the atomic model of sfCHT viewed from the membrane, colored in a rainbow gradient from blue (N-terminus) to red (C-terminus). The missing loop between TM helices 2 and 3 is depicted as a dashed line. Na^+ cation is shown as a purple sphere, and choline as sticks and colored by heteroatom. The rectangle in dashed lines highlight the location in the structure of the enlarged view of Na^+ (C). (**C**) Close-up view of the Na^+ cation, with interacting residues depicted in sticks and the water molecule as a sphere.

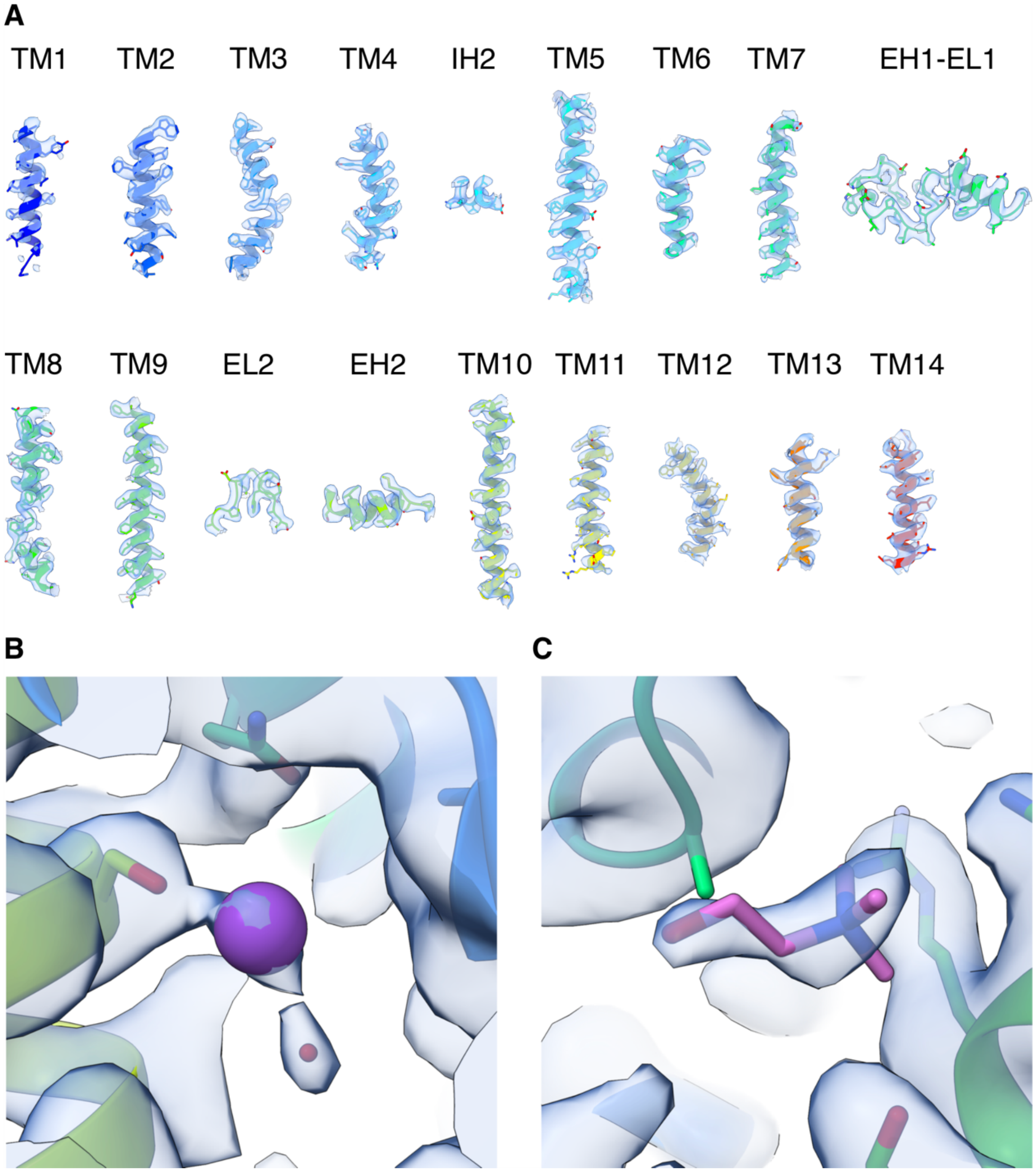

Fig. S12. Cryo-EM map and model-to-map fit of choline-bound sfCHT. The map threshold was set to an RMSD threshold of 6.30 Å in panel (A), and to 4.10 Å in (B) and (C). (**A**) The different segments of sfCHT, as named as in Supplementary Figure 1, are depicted in cartoon format with the side chains shown in sticks. The cryo-EM density map is displayed around these segments. (**B**) Close-up view of the Na^+ cation shown as spheres, interacting with residues depicted in sticks and the water molecule as a sphere. (**C**) Close-up view of the ligand choline bound near the intracellular side of the protein and the interacting ligands.

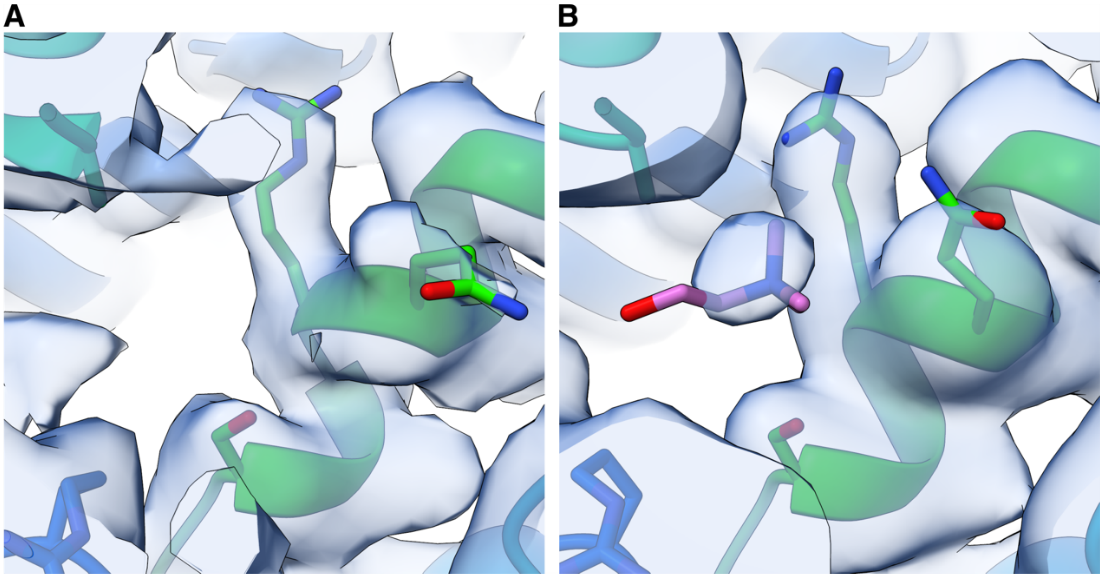

Fig. S13. Comparison of unsharpened cryo-EM maps and fitted atomic models Na^{+}-bound sfCHT (A) and choline-bound sfCHT (B) at the cytoplasmic site. The map threshold was set to an RMSD of 3.50 Å and 2.70 Å for (A) and (B), respectively.

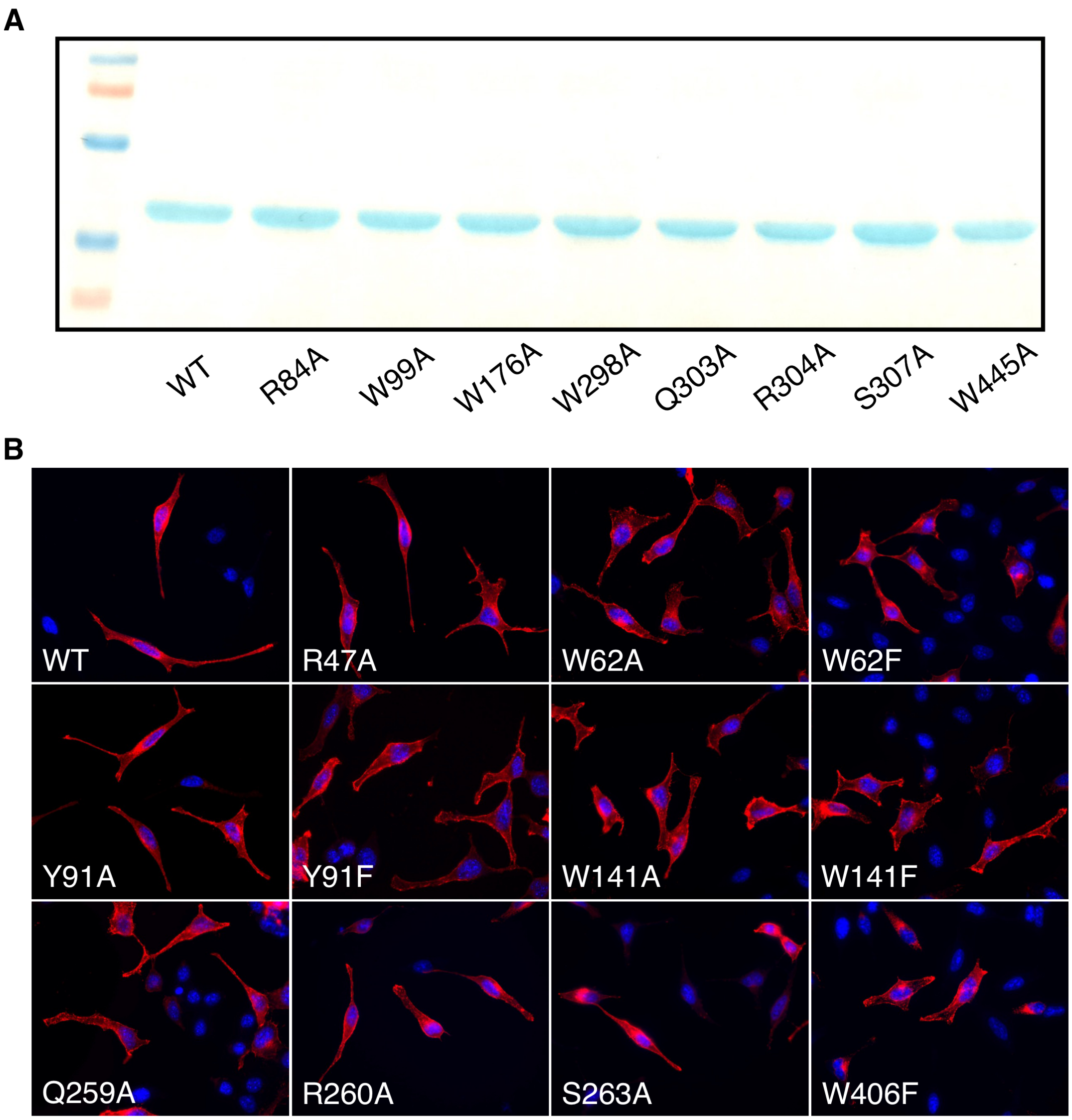

Fig. S14. SDS-PAGE analysis of liposome reconstituted WT and variant sfCHT (A) and expression of WT and variant CHT1 expressed in HeLa cells (B). Cells were seeded on 24-well plates, transfected and assayed 24 h post-transfection. Representative images of immunofluorescence of WT and variant CHT1. CHT1 (red) and Hoechst 33342 (blue) labelling is shown. All CHT1 variants were properly trafficked to the plasma membrane and display similar staining levels as WT CHT1.

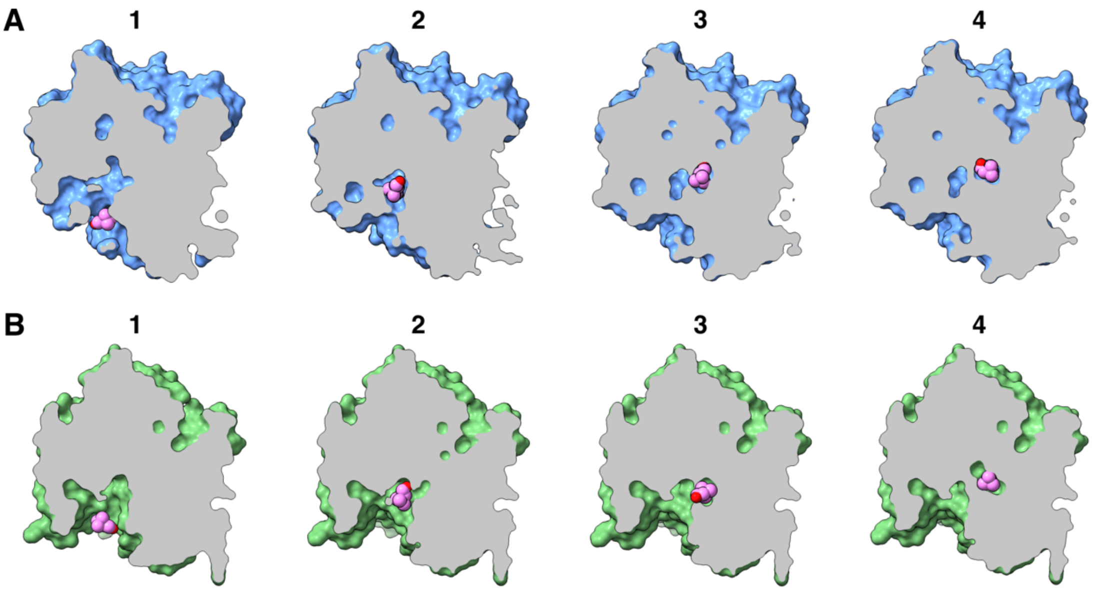

Fig. S15. **PELE-predicted choline poses along the intracellular tunnel**. Cross-sectional front views of sfCHT an CHT1 structures shown as surface representations. Choline molecules are displayed as spheres. (**A**) Choline poses along the intracellular tunnel in sfCHT. (**B**) Choline poses along the intracellular tunnel in CHT1.

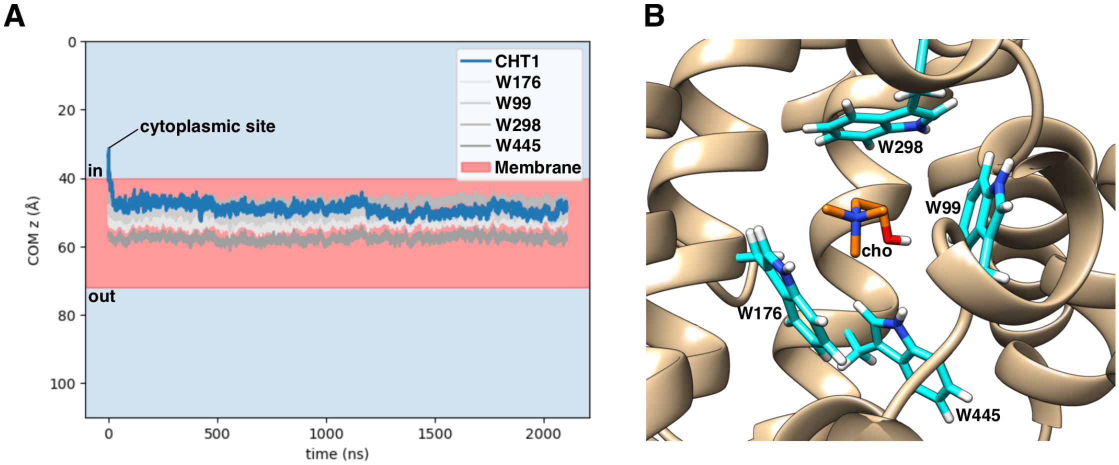

Fig. S16. **Molecular dynamics simulations under unbiased conditions**. (**A**) Time evolution of the z-axis position of the center of mass of choline and relevant tryptophan residues as a function of simulation time in nanoseconds. The background shading indicates regions inside the pore (red) and outside the pore (blue). Panel shows a representative trajectory from unbiased simulations. (**B**) Representative snapshot from an unbiased simulation showing the choline residue encapsulated within the pore by surrounding tryptophan residues.

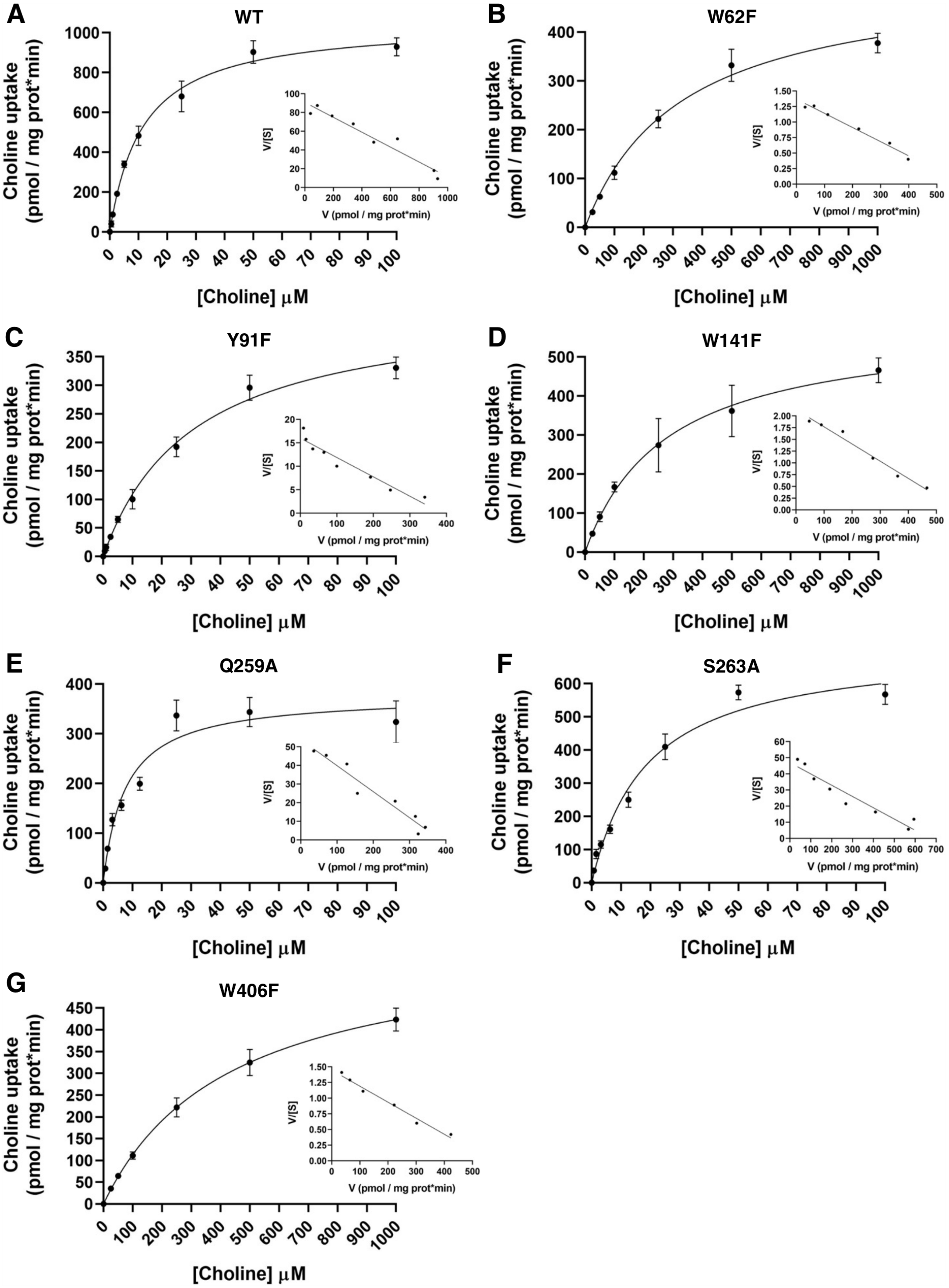

Fig. S17. WT and variant CHT1-induced uptake of [^{3}H]-choline kinetic analysis. Data (mean±SD) from representative experiments run in triplicates are shown. *Inset*: Eadie-Hofstee transformation.

Movie S1. Choline transition from the substrate binding site to the cytoplasmic vestibule. Morph with PELE predicted poses and the cryo-EM structure in the presence of choline.
(separate mp4 file)

Auxiliary Other Supporting file (tabulated data in Excel)

Data S1. Tabulated data for Figs. 1, 3C, 4B, 5B, 5C, S17. Data in tabular format used in Fig. 1, 3C, 4B, 5B, 5C, S17 with sample size N<20.
